# Integrated solutions for sustainable fall prevention in primary care: a pragmatic hybrid-type 2 mixed methods implementation and effectiveness study

**DOI:** 10.3389/fpubh.2024.1446525

**Published:** 2024-12-05

**Authors:** Lindy Clemson, Lynette Mackenzie, Meryl Lovarini, Christopher Roberts, Roslyn Poulos, Catherine Sherrington, Amy C. W. Tan, Judy Simpson, Constance Dimity Pond, Sabrina Pit, Anne Tiedemann, Lorraine Lovitt, Sarah N. Hilmer

**Affiliations:** ^1^Ageing and Health Research Group, The Faculty of Medicine and Health, The University of Sydney, Sydney, NSW, Australia; ^2^Sydney Medical School, The Faculty of Medicine and Health, The University of Sydney, Sydney, NSW, Australia; ^3^School of Population Health, The Faculty of Medicine & Health, University of New South Wales, Sydney, NSW, Australia; ^4^Sydney School of Public Health, The University of Sydney, Sydney, NSW, Australia; ^5^Discipline of General Practice, The Faculty of Health, University of Newcastle, Newcastle, NSW, Australia; ^6^University Centre for Rural Health, The University of Sydney, Lismore, NSW, Australia; ^7^School of Medicine, Western Sydney University, Lismore, NSW, Australia; ^8^Clinical Excellence Commission NSW, Sydney, NSW, Australia; ^9^Kolling Institute, Northern Sydney Local Health District and the University of Sydney, Sydney, NSW, Australia

**Keywords:** general medical practice, family practice physicians, allied health, cluster randomized controlled trial, surveys, mixed-methods, parallel study design, fall prevention

## Abstract

The iSOLVE implementation project established and evaluated integrated processes and pathways, including a decision-making tool and educational interventions for general medical practitioners (GPs) and the upskilling of allied health professionals (AHPs). The study used a mixed-methods (parallel) design comprising surveys, qualitative methodologies, and an embedded cluster randomized controlled trial (RCT). Sampling was conducted within a Primary Health Network (PHN) geographic area in Sydney, Australia. AHP workshops (*n* = 367 attendees) covered six evidence-based interventions, resulting in increased confidence (*p* < 0.001) and numerous enhancements in fall prevention delivery. Among GPs, 75 were recruited from 27 practices. GPs in the experimental group were more likely to engage in fall prevention activities, including risk assessments, medication reviews, and providing advice, compared to the control group (*p* = 0.002). They were also more likely to refer patients to AHPs at 3 months (*p* = 0.002); however, this effect was not significant at 12 months (*p* = 0.13), as referral behaviors increased in the control group over time. Responses to free-text questions of practice change highlighted differences, with the experimental group reporting a more proactive and comprehensive approach to fall prevention. In a subset of GP patients (*n* = 560), no significant effect was observed in reducing the rate of falls (IRR = 0.96). The pragmatic nature of the project and potential contamination across multiple elements likely influenced this outcome. However, an area-wide survey of GPs (*n* = 562) revealed an increase in fall prevention referrals to AHPs over 5 years, from 70 to 82% (*p* = 0.028). Our findings highlight the importance of equipping GPs with tools and strategies to adopt a proactive approach to fall prevention among older patients. AHPs play a crucial role in this effort, and fostering relationships and connectivity across primary care networks is essential to maximizing the impact of fall prevention initiatives.

**Clinical trial registration:**

Australian New Zealand Clinial Trials Registry, ACTRN12615000401550, https://www.anzctr.org.au/Trial/Registration/TrialReview.aspx?id=368286.

## 1 Introduction

It is estimated approximately a third of people over 65 years will have at least one fall per year (1), with more falls experienced by people over 75 years and some falling multiple times in a year. Primary care, which can include general medical practice, allied health services, community health, and community pharmacy, is generally the first point of contact people have with a health system. General medical practitioners (GPs) (family practice physicians), in particular, are relied on to manage the needs of older patients who experience falls (2, 3). This leaves many older people dealing with the aftermath of a fall in the community who could benefit from primary care interventions to prevent further falls. Falls are serious events with consequences of injury affecting mobility and independence, as well as psychological consequences such as loss of autonomy, loss of self-efficacy, and fear of falling (4), all of which would benefit from interventions through primary care (3).

Several Cochrane reviews have confirmed that exercise (5), reducing environmental fall hazards at home (6), and multi-component fall prevention (7) reduce the rate of falls in older people. Studies have also suggested that community fall prevention programs can reduce fall-related health service use (8, 9). A systematic review and meta-analysis of fall prevention by GPs did not demonstrate an effect on fall reduction, though it demonstrated an effect on injury prevention (10). The authors noted that the fidelity of interventions was limited by independent GP decisions and a reliance on patients to initiate intervention.

Despite strong evidence to guide effective fall prevention interventions in community-residing older people, there are few models (10, 11) and no clear model in Australia for engaging GPs in fall prevention. Additionally, routine use of allied health professionals (AHPs) in fall prevention has been slow, limiting widespread dissemination. To address these gaps, we developed the Integrated Solutions for Sustainable Fall Prevention (iSOLVE) implementation project (12) to establish and evaluate processes and pathways to identify at-risk older people and engage a whole primary care approach to fall prevention. We sought to engage GPs and AHPs in fall prevention, increase awareness, improve access to evidence-based fall prevention interventions, and enable ongoing knowledge acquisition and sustainable action. This study aims to describe how a multifaceted fall prevention process, implemented through upskilling in evidence-based practice and supporting workflow practices, can impact GPs’ and AHPs’ engagement in fall prevention for older adults in the community.

Our implementation objectives and related *study design characteristics* were to:

- Develop a process for implementing fall prevention in GP practice (*Developmen*t).
- Upskill AHPs within the Primary Health Network (PHN) geographic area in evidence-based fall prevention (*complementary upskilling*).
- Recruit, upskill, and engage recruited GPs in fall prevention management as a routine practice and evaluate the degree of practice change over 1 year [*sampling-cluster randomized controlled trial (RCT)*].
- Evaluate a subsample of GP patients’ engagement in fall prevention and effectiveness on the rate of falls over 1 year (*sampling-cluster RCT*).
- Explore GP and AHP experiences in iSOLVE (published elsewhere) (13–15) (*Convergence*).
- Evaluate engagement in fall prevention by GPs across the PHN geographic area over 5 years to explore flow-on effects from project activities *(Diffusion)*.
- Review drivers of practice change and develop online resources enabling education and support for sustained implementation of iSOLVE into primary practice *(Reflection and Expansion).*

## 2 Materials and methods

Table 1 outlines the objectives and descriptors of this pragmatic hybrid-type-2 effectiveness and mixed-methods (parallel) study (16, 17) to implement iSOLVE. Methods and results are presented for each objective.

**Table 1:** Objectives of the pragmatic iSOLVE project and descriptors of the (parallel) mixed-methods design.

Implementation research objective	Mixed-methods design	Sample	Data collection
1. Describe the processes of adapting and developing the components of a systems process for implementing fall prevention in GP practice	*Development* Consultations Resource development	Partnership with Primary Health Network (PHN); Advisory Panel. Decision tool – fall research expertise (LC, CS, AT); support from STEADi developers^a^ (11, 25)	Review and feedback on resources and processes developed by the iSOLVE team Decision tool – Expert review
2. To evaluate knowledge translation of evidence-based fall prevention by AHPs^b^ within the PHN^c^ who attend offered workshops.	*Complementary upskilling* AHP fall prevention workshops	AHPs within the PHN^c^–newsletters invitations to known providers and those nearby to recruit GPs.	Engagement in fall prevention practice and change over time—pre-post surveys at baseline, 3 and 12 months.
3. To evaluate the extent to which iSOLVE was effective in upskilling and engaging GPs in fall prevention management as a routine practice. 4. To evaluate patient engagement in fall prevention and the effectiveness of their rate of falls in a sub-sample of patients.	*Sampling* Cluster randomized controlled trial	Recruit GP practices and GPs within the PHN^c^. Provide educational details to GPs so they can introduce the iSOLVE process and resources. Recruit a subsample of GP patients.	Observations and reflections of GP engagement (detailing). GP practice change comparing experimental and control – surveys (baseline, 3- and 12-months); open-ended question – content analysis. Patient engagement in fall prevention – surveys (baseline, 3- and 12-months); fall outcomes by monthly fall diaries.
5. To explore experiences in embedding the process into usual care over time.	*Convergence* Interviews with participating GPs and AHPs	Interviews with experimental trial GPs (15) Interviews with AHPs who attended workshops (14)	Embedding fall prevention into usual practice in primary care –in-depth interviews (results published elsewhere) (13–15).
6. To evaluate engagement in evidence-based fall prevention across the PHN^c^ and explore if there are any flow-on effects from project activities.	*Diffusion* Area-wide survey	Area-wide annual survey of GPs within the PHN^c^.	GP practice change across the five-year project.
7. To identify factors that influence the embedding of iSOLVE into usual care and to develop online resources that will enable education and support for sustained implementation of iSOLVE into primary practice.	*Reflection and Expansion* Team discussion and reflections Develop/update educational resources	iSOLVE team. Learning designer input.	Drivers of change reviewed. Online learning modules for general practice, case audits for GPs, and an online GP risk assessment tool.

^a^STEADi developers: Judy Stevens and Tiffany Shubert (11, 25). ^b^AHP, allied health professionals. ^c^PHN, primary health network.

A parallel relationship denotes that the samples for the qualitative and quantitative components are different but are drawn from the same underlying population. Integrating complementary multiple methods allowed us to provide a more nuanced understanding of the implemented iSOLVE’s strengths, weaknesses, and real-world implications (17, 18). We drew on Palinkas et al. (19) and Gilmer et al. (20) in describing the characteristics of the mixed-methods design. These studies provided a structure for how the implementation questions and corresponding methods related to and built on each other.

In conceiving and designing the project, we partnered with a primary care network, the Northern Sydney Medicare Local (NSML), which, within the 1st year, was restructured into Sydney North Primary Health Network (the PHN). PHNs were established by the Australian Government to localize and improve the provision, coordination, and navigation of the complex healthcare system. They have a role in increasing the efficiency of medical services and in providing education and networking opportunities for health professionals. The major restructuring of the PHN resulted in a larger study recruitment area, a change of leadership, and the loss of our initial network partner. The new leadership supported the project, and while initially focused on their restructuring, their engagement evolved over time.

The final reflection and expansion phase reviewed drivers of change and emergent findings to produce final resources and a GP online learning module. Expected drivers of change were initially theorized in our protocol paper (12) using elements of the knowledge-to-action framework (KAT) (21), Michie et al.’s Behavior Change Wheel (22), and supported by Lau et al.’s review (23). Reporting of our implementation outcomes has been guided by Lengnick-Hall et al. (24) and Curren et al. (16).

Ethical approval was obtained from the Human Research Ethics Committee of the University of Sydney (2014/316, 2014/848). All GPs, patients, and allied health professionals recruited in the cluster RCT and in-depth interviews were given the Participant Information Statement and provided written informed consent. Survey participants were given their information statement at the start of the survey, and the submitted survey indicated consent.

### 2.1 Development phase

We consulted widely in developing the iSOLVE systems and resources during the project roll-out. We worked with service coordinators and management at the primary care network (NSML and the PHN) and our advisory group (GP, consumer, physiotherapist, exercise physiologist, occupational therapist, pharmacist, podiatrist, nurse, and fall prevention champions). We further consulted with local GPs, geriatricians, a geriatrician-clinical pharmacologist, and an ambulatory care specialist. The developers (11, 25) of the STEADi primary care resources in the US shared their resources and their experience with the team.

The development phase focused on developing resources and tools (12) to provide a simple workflow system for identifying people at risk of falls and to initiate fall prevention in GP practice. The resources adapted or developed for iSOLVE are summarized below.

**Figure 1:**
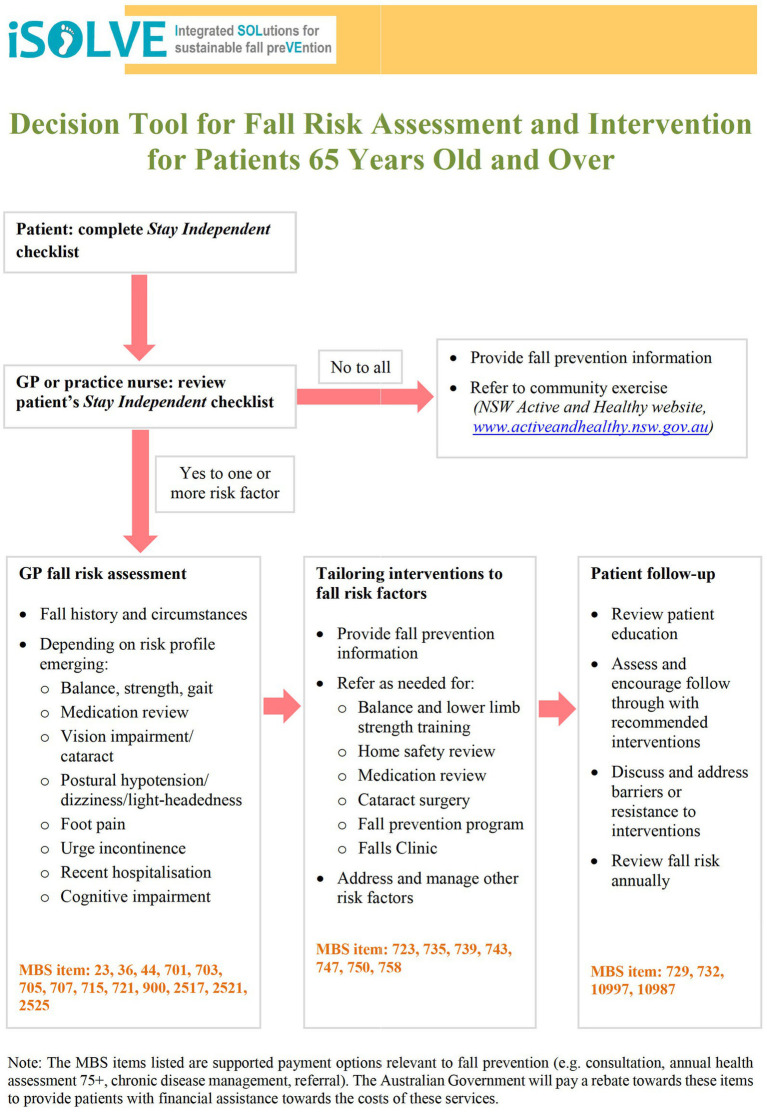
iSOLVE decision support tool for GPs (development reported in Clemson et al. (12) – see Table 1, column “Active ingredients”, section 1.2 in (12)). Decision tool and relevant resources are downloadable from Clemson et al. (27).

- *Decision Support Tool* to provide a simple workflow system for GP Practice, with the GP first asking the questions, ‘Have you had a fall?’ and “Are you worried about falling?.” The iSOLVE Decision Tool (Figure 1) was developed by a team of fall research experts (LC, CS, ATi) drawing on current evidence and based on the US STEADi algorithm (11, 26). The tool includes the patient Stay Independent checklist (Supplementary Appendix 1) and the GP Fall Risk Assessment (Supplementary Appendix 2). Along with a new element, we added ‘Tailoring Interventions to Fall Risk Factors’ (Supplementary Appendix 3) to map patient risk factors to appropriate interventions. Referral lists to local fall prevention service providers were also developed.
- *GP manual* (27) included summaries of fall prevention evidence, a series of case studies providing examples of using the Decision Tool and tailoring management options (Supplementary Appendix 4–example case study), Australian Government Medicare Benefits Schedule (MBS) funding options, and examples of ‘how to talk to your patients about falls’.
- *GP software.* We offered paper and electronic versions of the Decision Tool. The Decision Tool was developed within a commercial GP software (PenCS third-party software Topbar) that was used by some GPs in the area. A tablet, intended for practice nurses or reception staff use, was set up with a patient Stay Independent checklist to automatically send preliminary risk information to the GP software.
- GP face-to-face educational detailing sessions (2.3.1.1).

### 2.2 Complementary upskilling—AHP workshops

During the project, interactive workshops were delivered to educate and upskill AHPs in evidence-based fall prevention interventions. AHPs working within the PHN geographical area and providing services to older adults in the community were invited. Workshops were advertised by the PHN and to providers who advertised their services using publicly available databases. We also invited AHPs known to or located nearby to recruit general practices.

Participant AHPs were asked to complete an online survey pre-workshop and post-workshop at three and 12 months. Using mostly four-point Likert scales, the items related to the frequency of fall prevention screening, assessment, intervention and referrals, funding sources, beliefs about fall prevention, and confidence in fall prevention delivery. Changes in fall prevention knowledge were measured using a second survey related to each topic pre- and post-workshop. At follow-up surveys, questions included whether their fall prevention practice had changed and provided examples of changes and why change had not occurred. Frequencies and percentages were used to describe survey responses. The chi-square test was used to compare statistically. A content analysis (28) was conducted for the free-text practice change question. Similar responses were grouped into categories to analyze text data and then refined into subcategories.

Five workshop topics were offered (Table 2). The workshops comprised presentations of current evidence, case studies, and group discussions. Printed materials were provided, and workshops were recorded as webinars for future educational purposes (29). After each workshop, participants discussed strategies to implement and sustain fall prevention strategies in their workplace. All workshop leaders were academic and experienced researchers in their fall prevention specialty.

**Table 2:** AHP participant characteristics and workshop topics.

Workshop topics for preventing falls (duration), *n* = 367	Number of workshops (*n* attending^1^)
Exercise interventions (6 h) Functional Exercise (LiFE) (3 h) Foot and ankle interventions (3 h) Home environment intervention (3 h) Medication management (2.5 h)	3 (*n* = 60) 3 (*n* = 74) 2 (*n* = 119) 3 (*n* = 73) 2 (*n* = 41)

Workshop participants (baseline survey), *n* = 342	Number (%)
Profession	
Physiotherapist Occupational therapist Podiatrist Pharmacist Exercise physiologists Other/unknown	135 (40%) 82 (24%) 39 (11%) 36 (11%) 35 (10%) 15 (4%)
Service sector^2^	
Self-employed Private practice Public health service Private health organization Other	126 (37%) 91 (27%) 77 (23%) 73 (21%) 11 (3%)
*Employment status*	
Part-time/casual Full-time Contract basis Multiple employments Unknown	164 (48%) 155 (45%) 16 (5%) 2 (1%) 5 (1%)

Mapping for fall prevention referral, *n* = 123	Number (%)
Physiotherapist/exercise physiologist Occupational therapist Multi-AHP agency/outpatient services Fall prevention program Podiatrist/pedorthist Pharmacist	54 (44%) 16 (13%) 11 (9%) 6 (5%) 19 (15%) 17 (14%)

^1May attend one or more workshops. 2May work in more than one sector.^

Workshop participants also provided contact details if they wished to be linked to GPs for fall prevention referrals. A mapping exercise was undertaken, and an online database hosted by the PHN was created containing a list of fall prevention-trained AHPs by profession and area of service (30). This enabled local information to be provided for experimental GPs.

### 2.3 Sampling—cluster RCT

The cluster RCT (12) was conducted according to CONSORT guidelines for cluster randomized trials (31). Primary outcomes were GP fall prevention management practices and the effect of the rate of falls in a sub-sample of GP patients. Implementation outcomes included patient engagement in fall prevention. The sample size estimate of 560 patients from 28 GP practices, as reported in our protocol paper (12), was designed to have 80% power to detect a 15% between-group difference of falls based on previous meta-analysis (32) and expected loss of practices and patients. Recruitment strategies to engage general practices and GPs are described elsewhere (33).

#### 2.3.1 Engaging and upskilling GPs

GPs from the PHN were invited to participate and were expected to routinely assess and implement fall prevention with all their patients aged 65 and over. We aimed to recruit 28 GP practices. The general practice was the unit of randomization stratified by practice size (<80 eligible patients 65 years or older for low, ≥80 for high). Practices were randomized individually after completing GP recruitment within each practice [computer-generated block randomization at a distant site by a researcher (JS) not involved with allocation or data collection] (12). The research coordinator was blinded during GP recruitment but unblinded after randomization to recruit patients and implement interventions with GPs.

GP engagement in fall prevention management, including referral practices, measured the primary outcome of GP practice change. The unblinded research coordinator collected data by surveys at baseline, 3 months, and 12 months. The research team developed the GP survey (Supplementary Appendix 5) (academic GP, fall prevention, population health expertise; CR, CDP, LC, RP, SP) and piloted it with five GP colleagues.

##### 2.3.1.1 Educational detailing

Participating GPs from practices randomized to the experimental group were offered an educational detailing session (34, 35) conducted by ATa (pharmacist), who was trained in fall prevention. She was also involved in the development of the resources and participated in all AHP workshops. If randomized to the control group, the GP practice was offered the same educational detailing and resources after their 12-month follow-up. Observations and reflections of GPs’ engagement were reported in field notes following each session. Reflective analysis (36) included ‘thinking aloud’ and reflective recall by the facilitator (ATa) along with regular meetings with lead investigators (LC, LM).

A face-to-face format was chosen to train GPs on how to implement the iSOLVE Decision Tool in practice. A single one-hour session—either 1:1 or in a group session—was selected to recognize GP time constraints and supported in a Cochrane review (37). Sessions were also offered to practice nurses (if available) and other staff (if requested) to encourage a whole-practice approach.

Content covered instructions and background using the GP Provider Resource (27) and the iSOLVE Decision Tool, utilizing the iSOLVE case studies as illustrative examples and, if using, demonstrating the Topbar software; local referral options; discussion of overcoming challenges and implementation ideas; and opportunity to ask questions. The practice was provided with hard copy resources for GPs/practice nurses and the Stay Independent checklists (hard copy and tablet for those with Topbar) in the waiting room. A follow-up phone call was conducted with the GP or practice manager/nominated nurse 2–4 weeks after the session to allow for further questions. GPs interested in using the clinical audit activity for Continuing Professional Development points were followed up to assist with completing the activity based on their own patient consultations.

##### 2.3.1.2 GP trial survey: practice change and engagement

###### 2.3.1.2.1 GP practice change

At three and 12 months, GPs were asked a dichotomous (yes/no) question about changing how they managed their older patients who had fallen in the past 3 months. The data were first analyzed using logistic regression, considering clustering by general practice using a random effect and stratification by the practice size (note that this gives the *p*-value for comparing groups). The clustering by practice was small (ICC = 0.10) and not significantly different from 0 (*p* = 0.27) at 3 months, and ICC was 0 at 12 months. Modified Poisson regression with robust standard errors, allowing for stratification, was used to estimate the rate ratio for experimental participants compared with controls.

Those who responded yes to changing practice were asked for example(s) of how their practice had changed. We asked all GPs about challenges in doing fall prevention in their practice and to provide an example(s). Responses to open-ended questions were analyzed by content analysis and grouped thematically (38).

###### 2.3.1.2.2 GP engagement in assessing fall risk, reviewing medications, and providing advice

Engagement was determined by the survey questions: How many of your older patients (aged 65 years and over) do you (i) assess for fall risk factors, (ii) review medications likely to contribute to fall risk, and (iii) give verbal or written advice on ways to reduce fall risks? Responses were none, very few, some, or most; they scored 0 to 3, with the three questions summed to give a total out of 9. The total score at each follow-up was compared between groups using linear regression analysis with baseline score as a covariate and adjusting for clustering by GP practice and for stratification.

###### 2.3.1.2.3 GP engagement in referring for managing falls

GP referral behavior for managing falls was evaluated by asking to whom you refer your older patients because they are at risk of falling (never, rarely, sometimes, or often). A referral score was created by counting the number of types of practitioners (out of 13 possible professions) to whom a GP sometimes or often referred older patients at risk of falling. Because the distribution of referral scores was approximately normal at each time point, linear regression was used instead of the planned Poisson regression. The referral score for each GP at each follow-up was compared between groups using linear regression analysis with baseline referral score as a covariate and adjusting for clustering by GP practice and for stratification.

#### 2.3.2 Recruitment of subsample of patients

To recruit a sample of patients, the practice staff of participating GPs was asked to generate a list of patients 65 years and older. GPs were allowed to vet the list and exclude ineligible patients (unstable medical condition, moderate–severe dementia, receiving palliative services). The letter, signed by the GP, invited participation in the trial if they had had a fall in the past year or were concerned about falling. It included the study team’s contact details. We aimed to recruit 20 patients from each practice, for a total of 560. A research assistant (RA) blinded to GP practice allocation explained the study, obtained written consent, and conducted baseline assessments during a home visit. The participating patients from practices randomized to the experimental group were asked to make an appointment to see their GP to talk about preventing falls. They were given the patient Stay Independent checklist to complete before the visit and a copy of a fall prevention book (39). Patients from practices randomized to control were asked not to disclose study participation to their GP. GPs were not informed which patients were recruited to support the intended practice-wide adoption of the fall prevention process.

Baseline data included fall history, comorbidity, and number and type of medications. During the 12-month follow-up, patient participants were asked to self-report any falls in a monthly diary that was mailed to the blinded RA. After 12 months, a survey was mailed asking about their engagement in fall prevention and administered by phone if not returned. Control patients were provided with resources after 12 months (and after their GP received educational details) and asked to consult with their GP.

##### 2.3.2.1 Patient–participant interaction with GP, engagement in fall prevention, fall outcomes, and medication changes at 12 months

Patient-participant engagement in fall prevention was determined by asking, “Did you do any of the following in the past year to help you prevent falls?” with a list of possible actions. They were also asked, “In the past year, have you changed the way you do things to prevent falls?.” To determine interactions between them and their GP, they were asked if “In the past year, (i) has your GP asked you about your fall history?, (ii) have you talked to your GP about what you can do to prevent fall(s)?, (iii) did your GP provide any fall prevention advice?, and, (iv) did your GP refer you to anyone else for fall prevention?” The percent of patients responding yes was analyzed using mixed-effects logistic regression, taking into account clustering by both GP and general practice using random effects and stratifying by practice size.

To analyze total falls per patient-participant, we used negative binomial regression with days in the trial as the exposure, adjusting for stratification by practice size (low/high) and allowing for clustering by GP using the generalized estimating equations (GEE) approach with an exchangeable covariance matrix and robust standard errors. To examine clustering, a mixed-effects negative binomial model was fitted, allowing for clustering by both practice and GP within practice. This analysis showed that, after accounting for clustering by GP, there was no additional clustering by practice. To analyze medication changes, based on self-reported data, we evaluated changes in the prevalence of fall risk-increasing drugs (40) from baseline to 12 months.

### 2.4 Convergence—GPs and AHPs in-depth interviews

Cluster RCT experimental GPs were invited to participate in audiotaped interviews lasting 10–45 min about their experience of the iSOLVE process. Data were coded, and a thematic analysis of interview transcripts was conducted (41). AHPs who had attended iSOLVE workshops were invited to participate in audiotaped interviews to explore how fall prevention was being incorporated into their routine practice. Again, thematic analysis was used to analyze transcripts (41). These studies have been published elsewhere (13–15).

### 2.5 Diffusion—area-wide annual GP survey

The GP area-wide survey, replicating the trial survey (2.3.1) and piloted with 31 GPs in 2015, examined referral patterns and practices as markers of uptake and implementation across the PHN geographic region. Using the PHN GP database, the survey was mailed to a random sample of GPs each year for the first 3 years (25% in 2016; 50% in 2017 and 2018), and in the final year (2019), it was sent to all GPs. Chi-square tests were used to compare responses to questions about GP engagement in fall management and referral behavior over time. The last survey included an open-ended question related to change in practice, which was explored using content analysis (38).

### 2.6 Reflection and expansion

In the project’s final phase, the research team undertook a reflective review of the processes and the findings against the theoretical frameworks underpinning the planned active ingredients of the iSOLVE components (12). This enabled the mapping of elements to effective implementation of change strategies. This review was important as it brought together the team’s collective understanding of the mixed methodologies and helped make meaning of the findings (42). From this process, we determined what drivers of change should be replicated and how the resources developed during the project should be accessible in the future. As part of this process, the team worked with a learning designer experienced in developing teaching and learning products in higher education to translate the iSOLVE process and resources into an online format.

## 3 Results

**Figure 2:**
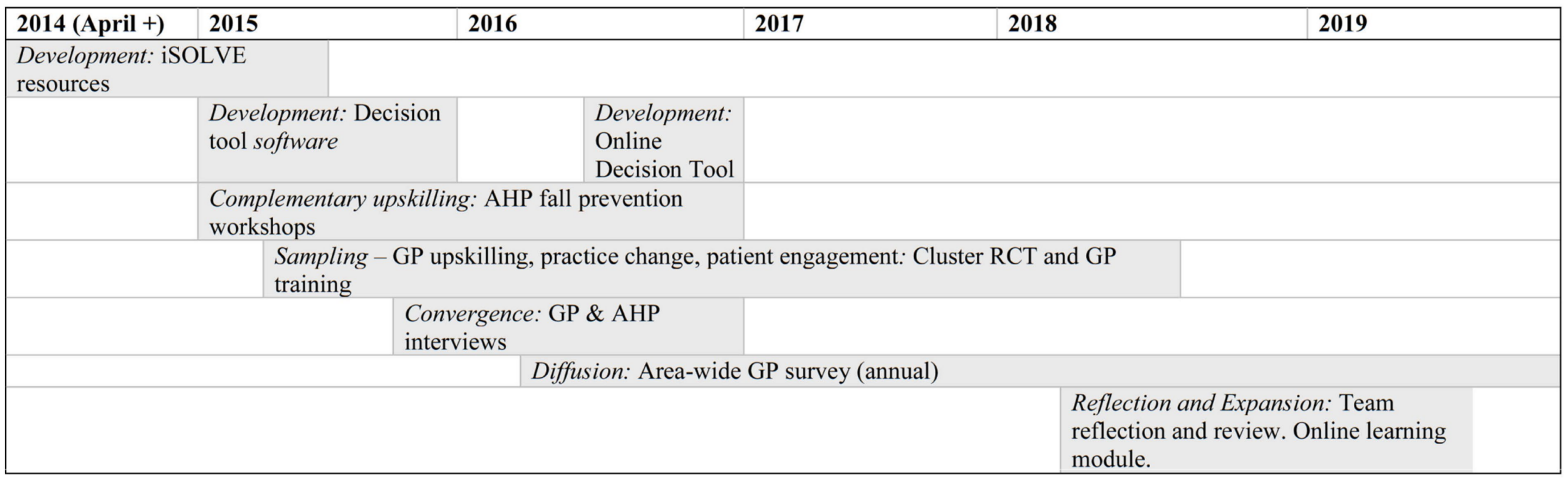
Timeline for the iSOLVE project.

The results are presented by the project implementation objectives as in Table 1. Figure 2 provides a timeline of the project over the 5 years.

### 3.1 Complementary skills—upskilling AHPs

#### 3.1.1 AHP workshops

Table 2 summarizes workshop topics, AHP-participant characteristics, and mapping for GP referral. The results from the pre-workshop and post-workshop knowledge questions are presented in Supplementary Appendix 6.

Of the 367 workshop participants, 342 of them submitted the baseline surveys (administered pre-workshop). Baseline survey responses showed that most were physiotherapists (*n* = 135, 40%), followed by occupational therapists (*n* = 82, 24%). The majority of the participants were self-employed (*n* = 126, 37%), with almost half (*n* = 164, 48%) working part-time or casually. At baseline, AHPs (*n* = 255) reported the number of referrals they received from GPs varied: 27% never or rarely, 43% sometimes, and 29% often. There was a large and diverse range of referral sources that included other AHPs (percent of sometimes/often: physiotherapists 59%, occupational therapists 53%, exercise physiologists 16%, podiatrists 9%, pharmacists 6%), fall prevention program (*Stepping On*) leaders (13%), falls clinic (25%), nurses (46%), and geriatricians (44%). They also frequently received self-referrals (67% sometimes/often).

#### 3.1.2 AHP-participant surveys at follow-up

Survey findings are presented in Table 3. Follow-up surveys were returned by 250 (73%) AHP participants at 3 months and 214 (63%) at 12 months. There was a statistically significant increase in self-confidence compared to baseline across all workshops. There was high engagement of AHPs in the frequency of fall prevention practices at baseline, with little change over the follow-up period in the frequency of fall prevention screening, assessment, and interventions. However, at 3 months, 74% of AHPs reported change in practice, and 66% reported change since baseline at 12 months.

**Table 3:** Allied Health Professional (AHP) outcomes from attending fall prevention workshops.

AHP changed practice as a result of the workshop				
	3-month		12-month	
All workshops	Yes % (*n*)	No % (*n*)	Yes % (*n*)	No % (*n*)
	74% (176)	26% (63)	66% (126)	34% (66)

Frequency of AHP engagement in fall prevention screening, assessment, and interventions						
Fall prevention activity	Frequency	Baseline % (*n*)	3-month % (*n*)	12-month % (*n*)	Chi sq (df) Baseline to 3-month	Chi Sq (df) Baseline to 12-month
Screening (all workshops)	Sometimes/Often Never/Rarely	78% (264) 22% (73)	82% (203) 18% (45)	82% (173) 18% (39)	χ^2^ (1) = 1.10, *p* = 0.30	χ^2^ (1) = 0.86, *p* = 0.36
Assessment (all workshops)	Sometimes/Often Never/Rarely	86% (289) 14% (48)	88% (217) 12% (31)	85% (180) 15% (31)	χ^2^ (1) = 0.37, *p* = 0.54	χ^2^ (1) = 0.02, *p* = 0.88
Intervention (all workshops)	Sometimes/Often Never/ Rarely	88% (296) 12% (41)	91% (224) 9% (22)	89% (188) 11% (23)	χ^2^ (1) = 1.53, *p* = 0.22	χ^2^ (1) = 0.20, *p* = 0.65

Improved AHP confidence in engaging in fall prevention						
Workshop topic	Confidence level	Baseline % (*n*)	3-month % (*n*)	12-month % (*n*)	Chi sq (df) Baseline to 3-month	Chi Sq (df) Baseline to 12-month
All workshops	Quite/very Little/not	60% (199) 40% (132)	85% (210) 15% (38)	87% (182) 13% (28)	χ^2^ (1) = 41.22, *p* < 0.001	χ^2^ (1) = 43.47, *p* < 0.001
Exercise	Quite/very Little/not	72% (39) 28% (15)	91% (39) 9% (4)	98% (39) 2% (1)	χ^2^ (1) = 5.19, *p* = 0.02	χ^2^ (1) = 10.40, *p* = 0.001
LiFE exercise	Quite/very Little/not	79% (53) 21% (14)	93% (53) 7% (4)	85% (35) 15% (6)	χ^2^ (1) = 4.78, *p* = 0.03	χ^2^ (1) = 0.66, *p* = 0.42
Home environment	Quite/very Little/not	69% (44) 31% (20)	88% (37) 12% (5)	84% (31) 16% (6)	χ^2^ (1) = 5.27, *p* = 0.02	χ^2^ (1) = 2.77, *p* = 0.10
Foot and ankle	Quite/very Little/not	42% (46) 58% (64)	78% (63) 22% (18)	83% (59) 17% (12)	χ^2^ (1) = 24.62, *p* < 0.001	χ^2^ (1) = 30.19, *p* < 0.001
Medication management	Quite/very Little/not	47% (17) 53% (19)	72% (18) 28% (7)	86% (18) 14% (3)	χ^2^ (1) = 3.70, *p* = 0.05	χ^2^ (1) = 8.29, *p* = 0.004

Types of changes made by AHPs	
*Theme*	Examples of responses
Enhanced exercise interventions	Increased accuracy of balance testing, including new challenges. Emphasis on speed, challenge of balance exercises, balance and foot and ankle exercise, functional exercise.
Interactions with clients	Asking more questions. Use the health change model to assist the client in working out what they need to change to improve their safety.
Screening and risk assessment	More active and comprehensive. Used quicker balance tests from the course. A broader look at environmental hazards.
Working with other health professionals	As an organization working around a home-based assessment. Incorporating home environment as well as exercise.

Responses to the practice change question demonstrated evidence of change in the nuances and nature of how the AHPs delivered their fall prevention. For example, they broadened their awareness of fall prevention, added practice enhancements with new ideas, and referred to others to more fully meet clients’ needs, as illustrated below and in Table 3.

“Included more information in the assessment and have tried making the client more involved in finding solutions to the risks/issues/behaviors—asking more open-ended questions to guide the client in coming up with solutions him/herself (taking more responsibility in the process) and in setting goals.” (H3).

AHPs who did not change their practice cited issues such as implementing the interventions already or lack of time and opportunity (e.g., no referrals, inability to provide multiple visits, difficulty in providing a thorough service within funded appointment time, no older clients).

### 3.2 Sampling—cluster RCT

#### 3.2.1 Recruiting and training GP practices and GPs

**Figure 3:**
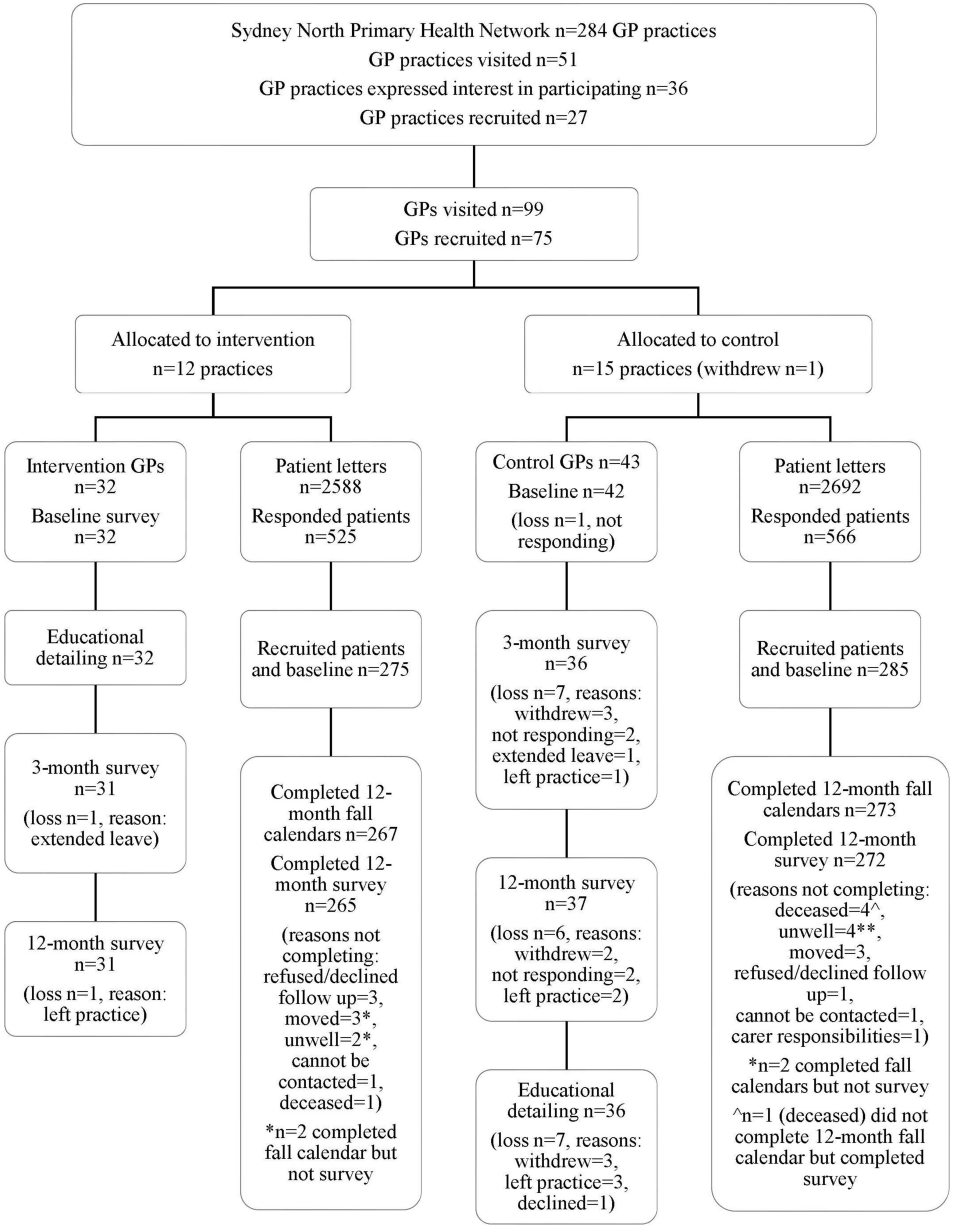
A flow chart of GPs and GP patients in cluster randomized controlled trial.

Twenty-six practices were randomized into two groups: 12 to experimental (32 GPs) and 15 to control (43 GPs). One practice (3 GPs) withdrew post-randomization. Figure 3 shows the cluster RCT flowchart and baseline characteristics of GPs, which are reported in Table 4 [Supplementary Appendix 7 reports the GP sample per GP practice and stratum (high/low)].

**Table 4:** Baseline characteristics of GPs (*n* = 75).

	Experimental *n* (%)	Control *n* (%)
Randomization by stratification of GP practice		
Low numbers expected <80 eligible patients	2 (6%)	7 (16%)
Higher numbers expected > = 80 eligible patients	30 (94%)	36 (84%)
Years practicing as a GP		
<5 years	2 (6%)	2 (5%)
5–10 years	4 (13%)	3 (7%)
>10 years	26 (81%)	38 (88%)
GP’s estimated percentage of patients 65 years and over		
<21%	5 (16%)	15 (35%)
21–40%	14 (44%)	19 (44%)
41–60%	8 (25%)	8 (19%)
61–80%	5 (16%)	1 (2%)

##### 3.2.1.1 Educational detailing

A total of 27 face-to-face sessions were conducted with 12 experimental GP practices. The 27 educational detailing sessions were attended by 32 GPs, two GP registrars, one medical student, and 13 practice nurses. There were 16 individual sessions, six with two participants and five with 3–5 participants. Of the 27 sessions, 17 were with GPs, seven were with practice nurses, and three were mixed staff sessions. The mean session duration was 51 min (range 30–90). The two sessions, which were 90 min long, were large practices with all recruited GPs in attendance. Eleven GPs chose to additionally undertake the case audits during the trial, with the PHN facilitating their professional education points.

A major part of the educational detailing was an emphasis on the GP’s role in routinizing fall prevention into everyday practice with every patient 65 years and over (i.e., asking if they have had a fall or worried about falling) and working through several iSOLVE case studies (27) (Supplementary Appendix 4), which demonstrate how the Decision Tool is used and how to tailor fall prevention interventions. As an alternative to prepared cases, some GPs reflected on their own patient(s). A common theme raised by GPs was their referral practices and familiarity with local fall prevention providers; local referral lists to AHPs were well-received. Another common theme was patient resistance or systemic difficulties in accessing services; counterpoints (e.g., how to encourage patients, transport options) were offered to assist GPs with their patient consultation.

The majority of the recruited GPs appeared keen to learn and felt it aligned with their vision of wellbeing for their older patients. Some GPs could not believe that their patients were interested, particularly those they perceived as low or no risk. Peer influences were evident during discussions when some recruited GPs shared resources with others in practice, and six practices requested additional education be organized for new GPs or staff.

Six practices had ‘top-up’ sessions to demonstrate the GP software, which was delayed in development. Some GPs opted to stay with paper versions; others experienced some software function and practice computer issues. During the trial, the team developed an external web-based decision tool as an alternative that provided easier access for GPs to practice the decision tool.

#### 3.2.2 GP practice change: RCT survey

##### 3.2.2.1 Changed practice in managing older patients who have had a fall

Both groups reported change in practice. Experimental GPs were significantly (*p* = 0.04) more likely to have changed the way they managed their older patients who had had a fall and were 1.6 times more likely to have changed than those in the control group (Table 5). At 12 months, the risk ratio was smaller, though this may be due to the question only asking about changes in the past 3 months.

**Table 5:** GPs who changed the way they manage older patients who fall and types of changes made.

GPs who changed the practice				
	Yes changed practice		Risk ratio (95%CI)	*P*-value
	Experimental	Control		
3-month	25/31 (81%)	18/36 (50%)	1.59 (1.09, 2.31)	0.04
12-month	22/31 (71%)	18/37 (49%)	1.41 (0.94, 2.11)	0.09

Types of changes made by GPs		
*Theme*	Experimental GPs’ responses	Control GP’s responses
Asking the question and identifying at-risk	‘More comprehensive in the assessment. Asking all over 65 s re: risks.’ ‘Finding the falls – often patients are reticent to notify us of falls ‘	‘More consistently assessing the risk of falling as part of a general review of older patients.’
Attention to risk screening and management	‘Specific questioning and completion of checklist for risk of falling. Review of medicines, questioning eyesight review, discussing exercise regime and other areas associated with fall risk such as foot care.’ ‘Using the iSOLVE tool has enabled me to more methodically assess my patients who have had a fall or feel they are at risk.’ ‘I ask more questions re eyesight, balance + BP medications and medication side effects in general.’ ‘Assess reasons for falls more effectively.’ ‘More frequent screening. A better understanding of contributing factors to falls.’	‘Earlier assessment, review of medications after excluding any acute problems.’ ‘Better history, assessment; medical approach; personalization of situation.’ ‘made sure has had a bone density in the past 2 years.’ ‘Increased use of home OT assessment.’ ‘Actively screen for foot/eye problems. Initiate more medication reviews. Refer to Falls Clinic more frequently.’
Referring on	‘More proactive in referring for falls prevention program.’ ‘I refer to active exercise classes, physios, and balance classes more often.’ ‘Refer to the *Stepping On* program.’	‘More likely to refer to Falls Clinic, physiotherapist.’ ‘Medication review with a pharmacist, involving other paramedical assessment (podiatrist, physio, OT review).’ ‘Sending more patients to see exercise physiologists.’
Engagement of practice nurse	‘Implemented comprehensive risk assessment Involved Practice Nurse in assessment.’	‘More targeted approach to therapy – nurse-led management’

For those who answered ‘yes,’ content analysis of their comments about how they changed practice revealed change across four themes: ‘*Asking the question and identifying at-risk*,’ ‘*Attention to risk screening and management*, ‘*Referring on,’* and ‘*Engagement of practice nurse*’ (examples in Table 5). While more experimental GPs reported change, both groups demonstrated change. Overall, those in the experimental group reported change across more preventive dimensions, often reporting a more ‘proactive’ and comprehensive approach involving discussion with their patients. The control GPs were most likely to focus on medical risk factors and predominantly on referral, which reflected their knowledge of fall prevention and increased knowledge of local referral options, particularly those listed in the survey. A few practices in both groups (six experimental, five control) involved their practice nurse implementing iSOLVE.

##### 3.2.2.2 Challenges to doing fall prevention

Content analysis of GP responses to challenges in doing fall prevention in their practice was explored in the context of ‘yes’ or ‘no’ answers to items indicating change in practice. In the experimental group, responses to challenges at each follow-up were provided by 17 who reported practice change and two who reported no change. There were an additional 12 GPs who reported practice change in one survey and no change in the other. In the control group, responses to challenges at each follow-up were provided by 13 GPs who reported practice change and 8 GPs who reported no change. An additional 16 GPs reported practice change in one survey and no change in the other.

The most common challenge theme for the experimental group that reported change in practice was *patient factors* (such as patient reluctance), which were closely followed by *time*. Other themes were *service issues*, and several GPs reported the *complexity/frailty* of their fall patients. For those experimental GPs who did not report a change in practice, the most common theme was *time*. The most common issues for the control group who reported change in practice were *patient factors*, followed by *comorbidity/frailty*, *service* *issues*, and *time*. These findings were similar for the control GPs, who reported no change in practice.

There were further differences, similar to the challenges between the experimental and control groups. It appeared that many experimental GPs practicing fall prevention at 12 months also recognized challenges that can make it difficult to be thorough. For example, one GP whose practice change was expressed as ‘*Assess reasons for falls more effectively*’ also noted challenges of ‘*Patients are often resistant to change (SIC) their behavior. Often not enough time to do a thorough job*.’ Control GPs tended to see multiple problems and more often associated falls with a person who, as one GP expressed, ‘*tends to be frail, older adult, has difficulty, and (therefore) is disinterested in participating in (fall) management*.’ Another responded, ‘*It is a multifactorial problem, often part of the complex clinical setting, and there are so many things to consider.*’ The experimental group tended to be more succinct and often focused on convincing their patients of the importance of fall prevention, as one GP stated, ‘*Convincing patients that they are a fall risk. Older adult men are not convinced until they have a fall*’. Time constraints for the experimental group were often mentioned as the ‘*time it takes’* or the time to conduct a ‘*full fall prevention.’* The control group saw more complexities around *accessing service providers* and were unsure of services, issues that were much less evident in the experimental group. The control group GPs more often listed *practical issues* of transport, home environment hazards, and mobility aids.

##### 3.2.2.3 Engagement in fall prevention: assessing fall risk, reviewing medications, and providing advice

A total of 66 participating GPs answered the three questions about assessing risk, reviewing medications, and providing advice at both baseline and 3 months. The model estimate of the engagement score was significantly higher (0.98 units, 95%CI 0.36 to 1.60) (*p* = 0.002) in the experimental group than the controls at 3 months, after adjusting for baseline score and stratification (see Table 6).

**Table 6:** Participating GP engagement in fall prevention.

GP Assessing risk, medication review, advice^1^								
	*n*	Baseline *M* (SD)	3-month *M* (SD)	*P*-value	*n*	Baseline *M* (SD)	12-month *M* (SD)	*P*-value
Experimental	31	6.58 (2.03)	7.00 (1.46)	0.002	31	6.58 (2.03)	7.55 (1.26)	0.002
Control	35	6.09 (1.93)	5.83 (1.36)		37	5.92 (1.85)	6.41 (1.46)	

GP referral practice for fall prevention^2^								
	*n*	Baseline *M* (SD)	3-month *M* (SD)	*P*-value	*n*	Baseline *M* (SD)	12-month *M* (SD)	*P*-value
Experimental	31	6.06 (2.87)	7.81 (2.82)	0.045	31	6.45 (2.98)	8.58 (2.78)	0.13
Control	35	6.17 (2.62)	6.66 (2.72)		37	6.22 (2.62)	7.43 (2.88)	

^1^Composite score (none to most (0–3)) of how often they engaged with older patients in managing falls, max 9. ^2^Composite score of ‘sometimes or often’ referred to a possible 13 types of practitioners/program. *n*, number who answered at both time points.

The estimated intra-cluster correlation (ICC) was 0, indicating no clustering by practice. Among the 68 GPs who responded to all three questions at both baseline and 12 months, the engagement score in the experimental group was significantly higher by 0.90 units (95%CI 0.33 to 1.46; *p* = 0.002) compared to the experimental group at 12 months. This result accounts for adjustments based on baseline scores and stratification, confirming the effectiveness of the intervention in improving engagement.

##### 3.2.2.4 Engagement in fall prevention: referral behavior

More than half of the GPs in the experimental group (55%) used the Active and Healthy Website to find local exercise and fall prevention classes at 3 months, an increase from 6% at baseline, and this was largely maintained at 12 months (42%). Usage by control GPs remained at only 2–3% over 3 months and increased to 14% at 12 months.

Table 7 outlines the practitioners and programs to which the GPs referred their older patients at risk of falls (% sometimes/often). Among the GPs who answered at both baseline and 3 months (Table 6), the model estimate of the referral score, out of a possible 13, was significantly higher (1.10 units, 95%CI 0.02 to 2.18) in the experimental group than the controls (*p* = 0.045) at 3-months, after adjusting for baseline score, clustering, and stratification. The estimated intra-cluster correlation was ICC = 0. Among the GPs who answered the question at both baseline and 12 months, the model estimate of the referral score was higher but not significant (1.29 units, 95%CI −0.38 to 2.97) (*p* = 0.13) in the experimental group than control at 12 months, after adjusting for baseline score, clustering, and stratification. In this case, there was clustering by practice, with an estimated ICC = 0.39.

**Table 7:** Referral by trial GP to practitioner/program: ‘Sometimes or often’ refer older patients at risk of falling.

AHP/Program	Group	Baseline		3-month		12-month	
		*n*	%	*n*	%	*n*	%
Physiotherapist	Experimental	26	81%	26	84%	30	97%
	Control	34	81%	31	86%	32	86%
Exercise Physiologist	Experimental	15	47%	22	71%	23	74%
	Control	22	52%	24	67%	26	70%
Occupational therapist	Experimental	17	53%	20	65%	21	68%
	Control	19	45%	21	58%	23	62%
Podiatrist	Experimental	21	66%	25	81%	26	84%
	Control	22	52%	22	61%	25	68%
Pharmacist	Experimental	18	56%	19	61%	26	84%
	Control	14	33%	19	53%	23	62%
Community exercise	Experimental	21	66%	27	87%	24	77%
	Control	28	67%	22	61%	29	78%
Stepping on fall prevention program	Experimental	9	28%	24	77%	24	77%
	Control	9	21%	10	28%	13	35%
Falls clinic	Experimental	16	50%	16	52%	18	58%
	Control	27	64%	23	64%	28	76%
Geriatrician	Experimental	23	72%	17	55%	19	61%
	Control	32	76%	27	75%	26	70%
Optometrist/Ophthalmologist	Experimental	7	22%	12	39%	16	52%
	Control	13	31%	9	25%	12	32%
Other specialist doctor	Experimental	9	28%	13	42%	12	39%
	Control	18	43%	14	39%	18	49%

Physiotherapists were the most common practitioners with high referrals at all time points. The three-month referral difference appears to be driven by a very large increase in referrals to the Stepping On fall prevention program and larger increases than controls in referrals to exercise physiologists, podiatrists, community exercise, and optometrists/ophthalmologists. At 12 months referrals across services were more balanced between the two groups, though for the experimental group still remained a higher proportion for Stepping On programs and for the control group a higher proportion to Fall Clinics, geriatricians, and other specialist doctors.

#### 3.2.3 Patient engagement in fall prevention, interaction with GP, fall and medication outcomes

In the experimental group, 275 patients were recruited from 30 enrolled GPs (mean 9.2 patients per GP, range 4–35). In the control group, 285 patients were recruited from 37 enrolled GPs (mean 7.7 patients per GP, range 3–35) (Supplementary Appendix 7—patient sample per practice).

Figure 3 shows a flowchart of patients in the cluster RCT, and Table 8 outlines the patient’s characteristics at baseline. There was a high percentage of patients (over 50%) who had a history of one or more falls in the past year, with half of these reporting multiple falls (50% experimental, 41% control), reflecting the subgroup of recruited patients who were at high risk of falling.

**Table 8:** Patient trial participant baseline characteristics.

	Experimental (*n* = 275)	Control *(*n = 285)s
Age [*M* (SD, range)]	78.5 (72, range 65–94)	78.6 (72, range 65–95)
Gender: Men (*n*, %)	93 (34%)	87 (31%)
One or more falls past the year (*n*, %)	153 (56%)	153 (54%)
The number falls past year [*M* (SD, range)]	1.05 (1.38, range 0–10)	0.96 (1.61, range 0–20)
Injurious falls in the past year (excludes bruising, cuts, and grazes) [*M* (SD, range)]	0.36 (0.60, range 1–2)	0.36 (0.68, range 1–3)
Fall-related hospitalization in the past year (*n*, %)	100 (36%)	122 (57%)
Use of walking aid (*n*, %)	92 (34%)	107 (38%)
Falls efficacy scale (65) [*M* (SD, range)]	11.41 (3.56, range 7–25)	11.68 (3.35, range 7–25)
Functional comorbidity index (66) [*M* (SD, range)]	3.63 (2.00, range 0–11)	3.80 (1.99, range 0–10)
Polypharmacy: 4 or more medications (*n*, %)	187 (68%)	193 (68%)

At 12 months, patients were asked what they had done in the past year to help them prevent falls (Table 9). Seventy-nine percent of experimental (*n* = 216, mean = 2.4, SD 1.5) and 69% of control patients (*n* = 196, mean = 1.6, SD 1.5) reported one or more fall prevention activities, the median of both being two and maximum six. Three types of activities indicated significantly higher engagement by experimental patients: prescribed home exercise, fall prevention program, and medication review. There was also a significant difference, with the experimental group being 1.9 times more likely to ‘change their ways’ to prevent falls.

**Table 9:** Participating patient engagement in fall prevention and fall-related interactions with GP, including referrals from GP.

Participating patient engagement	Experimental % of 275	Control % of 285	Odds ratio (95%CI)	*P*-value	ICC_GP_	ICC_pr_
Type of activity						
Group exercise, including balance	37	30	1.36 (0.79, 2.33)	0.26	0.076	0.037
Prescribed home exercises	55	41	1.74 (1.11, 2.73)	0.015	0.029	0.026
Fall prevention program	32	18	2.17 (1.40, 3.39)	0.001	0.016	0
Hazard review by occupational therapist	24	21	1.25 (0.81, 1.95)	0.32	0	0.008
Medication review by GP/pharmacist	70	55	1.86 (1.30, 2.68)	0.001	0	0
Cataract removal	17	16	1.18 (0.74, 1.89)	0.49	0	0
Attend Falls Clinic	6	6	1.24 (0.56, 2.74)	0.60	0	0.029
Changed ways to prevent falls	72	57	1.86 (1.26, 2.75)	0.002	0.014	0
Interactions with GP						
(i) GP asked about fall history	39	24	2.19 (1.44, 3.33)	<0.001	0	0.011
(ii) Talked to GP about falls	36	21	2.12 (1.31, 3.44)	0.002	0.034	0.027
(iii) GP fall prevention advice	43	20	3.15 (1.92, 5.19)	<0.001	0.048	0.015
GP referral to prevent falls	21	10	2.49 (1.24, 4.99)	0.01	0	0.080

The number of missing responses varied from 28 to 36 for the type of activity and 25–33 for the other items. ICC_GP_ intracluster correlation for clustering by GP. ICC_pr_ intracluster correlation for clustering by general practice.

Table 9 also shows the percentage of patients reporting interactions with their GP related to fall prevention, with the experimental group more likely to interact with their GP. Patient recall of referral by GP for fall prevention for both groups was low (10–21%), though significantly different between groups.

##### 3.2.3.1 Patient fall outcomes

There was a mean of 1.52 (SD 2.68) falls (range 0–27) in the experimental group and 1.67 (SD 3.70) falls (range 0–49) in the control group. There were 60% (*n* = 164) of patients in the experimental group who fell one or more times in the 12-month follow-up period, compared with 57% (*n* = 161) in the control group (Supplementary Appendix 8—total falls and time in a trial). The analysis showed no effect of the iSOLVE intervention in reducing the number of falls, with an estimated incidence rate ratio of 0.96 (95% CI: 0.77 to 1.20; *n* = 570). This means little or no difference in the rate of falls (4% reduction), with a wide confidence interval from 23% fewer falls to 20% more falls.

##### 3.2.3.2 Patient medication outcomes

The mean number of fall risk-increasing drugs at baseline was 2.1 ± 2.0 (control group) and 1.8 ± 1.6 (experimental group, *p* < 0.05). The mean change in the number of fall-risk-increasing drugs from baseline to 12 months was an increase of 0.11 ± 0.05 in the control group and 0.14 ± 0.04 in the intervention group (n.s.). The fall risk-increasing drug classes that were ceased most commonly were diuretics, calcium channel blockers, ACE inhibitors/angiotensin, and two receptor antagonists in both the experimental and control groups.

### 3.3 Convergence—in-depth interviews with GPs (experimental group) and AHPs

Interviews with the GPs in the experimental group (*n* = 24, 75%) enabled an in-depth perspective on their experience of embedding fall prevention in their everyday practice over time and reported elsewhere (15). Six themes were identified: (i) *making it easy to ask the iSOLVE questions, (ii) internalizing the process*, (iii) *integrating the iSOLVE into routine practice*, (iv) *addressing assumptions about patients and fall prevention*, (v) the *degree of change in practice*, and (vi) *contextual issues influencing uptake*.

In interviews with AHPs (*n* = 15) who attended the upskilling workshops, four major themes emerged and were reported elsewhere (14). These were (i) AHPs *valued fall prevention in practice as they recognized benefits for themselves and their clients*, (ii) AHPs recognized the *complexity of fall prevention work, such as complex clients, relationships with other health professionals, and changing funding environments*, (iii) AHPs *worked through complexities* according to client demographics and issues with running a business, and (iv) *strategies adopted for integrating fall prevention into routine practice* included asking every client about falls, being aware of falls as relevant to many clients, having processes in place to assess clients for risk of falls, and having a structured program for fall prevention.

### 3.4 Diffusion—area-wide annual survey 2016–2019

Response rates to the area-wide GP survey averaged 20–28% [2016 (*n* = 81), 2017 (*n* = 155), 2018 (*n* = 124)] for the first 3 years of sampling, and in the final year (2019) were 15% (*n* = 202) when sent to all GPs in the area. Surveys totaled 562 (overall response rate 20%) from 2,847 invitations. Across all surveys, 7% had <5 years practicing as a GP, 10% had between 5 and 10 years, and 83% had >10 years’ experience. Across all surveys, 20% of GPs estimated they saw less than 20% of patients 65 years and over, 53% estimated 20–40, 18% estimated 40–60, 8% estimated 60–80, and 1% of GPs had higher than 80% of patients 65 years and over.

#### 3.4.1 GP engagement in fall prevention, including referral behavior

GP engagement in assessing fall risk, reviewing medications, and providing advice did not significantly change from 2016 to 2019. Overall referrals for fall prevention to AHPs increased significantly from 70% in 2016 to 82% in 2019 (*χ*^2^ = 4.85, df = 1, *p* = 0.028). Type of referrals showed that referrals to Falls Clinics (*χ*^2^ = 6.02, *p* = 0.014, df = 1) and the *Stepping On Fall Prevention* Program (*χ*^2^ = 9.37, *p* = 0.002, df = 1) increased significantly, but others (physiotherapist, exercise physiologist, occupational therapist, community exercise class, and pharmacist) were unchanged. Reported familiarity with fall prevention services in the local area significantly increased from just over half (51%) in 2016 to almost three-quarters (72%) in 2019 (χ^2^ = 10.41, *p* = 0.001, df = 1), though the use of the NSW Health Department Active and Healthy website to refer to local exercise classes remained low (4–7%) (see Supplementary Appendix 9 for a full description).

#### 3.4.2 Changes in practice

In the final 2019 annual survey, a free-text question asked how GPs changed practice and the challenges they encountered. Changes in practice were analyzed in the context of the GPs’ confidence level in fall prevention. Half the GP respondents (*n* = 99, 49%) indicated changes had been made to practice, and all of them indicated some level of confidence: 29% rated themselves as having a little confidence, 62% quite confident, and 9% as very confident.

Five key themes emerged in the free-text responses relating to changes in practice: *medical interventions*, *fall prevention actions*, *referral behavior, attitudes to fall prevention*, and *social factors.* Responses varied markedly in those who reported increased confidence and are summarized in Table 10.

**Table 10:** Area annual GP survey (final year): key themes for change in practice and challenges.

Yes, changed practice (sample 2019 area survey, 49% *n* = 99)	
Themes	Summary of responses relevant to confidence
Medical interventions	Medical checks were identified and were more detailed by respondents reporting some confidence, although they were not identified as a change in practice by participants who rated themselves as being very confident.
Fall prevention actions	These were evident across the confidence continuum. This included reviewing fall risks, asking about falls, conducting fall assessments, and conducting closer patient follow-ups, with some using iSOLVE materials and/or integrated within the MBS 75+ health assessment.
Referral behavior	Referral behavior developed as confidence increased. This was done by referring more to targeted referrals and then to specific referrals (including community-based programs).
Attitudes to fall prevention	Attitudes shifted from being more aware to being more proactive in identifying risk earlier, managing falls, and discussing fall risk.
Social factors	These were considered an element of change in practice only by respondents who rated themselves as ‘little or quite confident.’ They were concerned with more general social issues, such as isolation and malnutrition, followed by involving friends and carers in fall prevention.

Challenges in providing fall prevention (sample 2019 area survey, *n* = 204)	
Themes	Examples
Time issues	*Time challenges* being a part time GP, health problems as a priority, time for fall assessments, logistics of longer appointments, follow-up, and nurse appointments.
GP beliefs about patients	Patients viewed falls as being a normal part of ageing, and, along with observed sedentariness, impacted compliance and adherence. Needing to convince patients about the benefits of fall prevention. Perceived their patients did not easily accept their fall risk, tended not to report falls, and were fearful of embarrassment.
Service issues	*Access to services.* Physical access to face-to-face activities, private service costs, lack of publicly funded services, inadequacies of the MBS chronic disease management funding schemes to subsidise AHP costs, waiting lists, and availability of AHP services for a referral. Transport was an issue for patients, and GPs commented on the lack of continuity of care and available services.
Other issues	Need for integrated software support, falls not being classed as a medical diagnosis, and the need to focus on high-risk patients and the challenges of patients with cognitive problems.

All 204 respondents commented on challenges to providing fall prevention, with themes of *time issues*, *GP beliefs about patients*, *service issues*, and *other issues* such as patient characteristics (Table 10). Those who were very confident and had made changes to their practice described being intentional about providing fall prevention for their patients, whereas less confident respondents who made changes tended to be less proactive and more often externalized the challenges (e.g., the patient being negative, lacking insight; no practice nurse support). Regardless of GP confidence levels or change in practice, dominant challenges were aspects of limited time to undertake fall prevention activities and beliefs about perceived patient acceptance of risk or reluctance to change. However, more confident people were proactive despite these and other challenges. Those who did not change practice for each level of confidence reported a much larger list of challenges than those who did make changes. A very small number of GPs who rated themselves as not confident (n = 4) made no changes to their practice in fall prevention. This group tended to be less positive about fall prevention and were not convinced it was beneficial.

### 3.5 Reflection and expansion

A reflective review by the research team of the iSOLVE process and findings enabled the mapping of iSOLVE elements to effective implementation change strategies (21–23), as presented in Table 11. This provides a better understanding of key elements driving change in practice. These learnings from the project were used to frame the approach in translating the iSOLVE resources online so they continue to be efficient, available, and accessible. Working with the PHN, the process was also embedded in their Health Pathways (43), a local online resource for general practice, and ongoing access to the AHP mapping (30).

**Table 11:** Key drivers of change.

Change strategy	Descriptors of iSOLVE elements
Environmental restructuring (22)	Decision tools Efficient and simple system It can be used repeatedly until routinized Embedded within GP software or paper version Generated or template referrals for fall prevention
Training and skills (21–23)	Up-to-date knowledge and resources Case-based online module
Enablement (22, 23)	Linking and mapping AHPs Communication and networking (GP/AHP; the PHN) PHN support for AHP workshops and promoting the iSOLVE project and activities across their network
Relationships (21–23)	Having the conversation – GP patients AHP: ‘You are not alone.’ PHN support in the accreditation of GP educational detailing and clinical audit activities
Reflective motivation (22)	Broaden GP focus to prevention Intentions, e.g., to observe the patient’s mobility as they walk in and sit down
Modelling (21, 22)	Relevant cases linked to fall risk decision tool Videos with key messages
Incentives (22, 23)	Funding options Easy access to online training module/decision tool Continuing Professional Development clinical audit points

References: Knowledge-to-action framework (21), Behaviour Change Wheel (22), review of factors influencing practice change in primary care (23).

As it was found overall that it was not workable to embed the Decision Tool in the commercial software (Topbar), the final product was an online website managed by the New South Wales State Falls Network. Thus, the planned dissemination document as per our protocol (12) was replaced by online resources for general practice, which include an interactive GP fall risk assessment (44) and downloadable paper versions of the patient Stay Independent checklist, GP fall risk assessment, and Tailoring interventions to fall risk factors (27). We also developed GP learning modules that provide an alternative to face-to-face educational detailing and a clinical audit activity eligible for GP Continuing Professional Development points (45). Online learning modules for AHPs are in development (45), and recorded workshops are available online (29).

## 4 Discussion

### 4.1 Our findings

This project was designed to influence clinical practice and address gaps in fall prevention across primary care, and our results were equally complex. Overall, our data provides insight into a number of mechanisms within a pragmatically implemented, multifaceted fall prevention process, which can improve GPs’ and AHPs’ engagement in fall prevention for older adults in the community.

The PHN area had a strong level of engagement by AHPs, particularly by physiotherapists, occupational therapists, exercise physiologists, pharmacists, and those delivering fall prevention group programs. This engagement was reflected by interest in both the workshops and mapping exercise, along with AHPs’ survey responses, demonstrating a high knowledge base. Post-workshop changes reflected increased confidence in fall prevention overall, and responses showed multiple enhancements in how they delivered fall prevention. The AHPs worked across sectors in the community, and referrals were from multiple sources, including GPs and between AHP disciplines. Our interviews with AHPs who attended the workshops (14) provide complementary evidence of how they valued fall prevention in their practice, recognized challenges in working alongside other health professionals, and appreciated a better understanding of others’ roles through the iSOLVE workshops. They reported concerns about complicated and changing funding systems impacting sustainable fall prevention. GPs value ‘reliable and good’ AHPs, with trust developing over time, though the patient more often delivers the feedback they receive than by the AHP (13).

We found GPs could change practice and engage in fall prevention. While both control and experimental GPs engaged in fall prevention, the experimental group was significantly higher at both time points for assessing fall risk, reviewing medications, and providing advice. Referral behavior was significantly higher for the experimental group at 3 months but not at 12 months, when the control group had increased their rate of referrals. Responses to free-text questions about practice change highlighted nuanced differences, with the experimental group reporting a more proactive and comprehensive approach compared to the control group, who were more likely to focus on medical risk factors and predominately on referral to specialists and Falls Clinics, reflecting their understanding of fall prevention. The GP software to support the GP management of patients at risk of falls did not work for all GP practices due to technical problems, but they could effectively use the paper alternatives (15).

Both experimental and control GPs reported challenges, in particular patient-related factors and time constraints. However, there were differences in the frequency and nature of how these challenges were perceived. Despite the challenges, more experimental GPs reported change in practice, and more often, they reported challenges centered around the thoroughness needed to identify and manage falls for their older patients. The control GPs focused on frailer patients with multiple problems, the complexities of accessing service providers, and practical issues such as transport. It appeared that their perceived role in the management of falls differed. A minority expressed that fall prevention was not worthwhile.

Interviews with experimental GPs (15) support and extend the findings of the GP trial survey. Groups that changed practice talked about how they engaged in the process as a simple’ system’ and provided ‘fall scripts’ to follow in what can be done and said. Over time, the process was internalized, where they asked their patients more in-depth questions. The opportunity to ‘practice’ with their at-risk patients was a process that appeared important in moving fall management to routine practice.

The outcome for the subgroup of GP patients did not translate to a significant increase or decrease in falls. The higher engagement of both control and experimental patient groups may reflect the area’s AHP activity, and contamination may occur when running an embedded trial in a broader implementation study. Engagement in fall prevention activities was moderate for both participating patient groups. However, the experimental group significantly engaged more often in interventions supported by robust evidence, that is, medication reviews by a GP or pharmacist (46), prescribed home exercises (5), and in a multicomponent fall prevention program (7). Our project provided extensive upskilling of AHPs in the study area who also reported multiple sources of referrals to them, including self-referrals. Our fall outcome was similar to the previous systematic review, (4) in which change in participant falls relied on GP referrals to others. While our experimental GPs engaged in broader fall management than referrals alone, the cluster RCT still relied on the small sample of participating patients initiating a referral with their GP. Recall of being referred for fall prevention by their GP was low (10–20%) in both groups. The Mackenzie et al. systematic review demonstrated a significant reduction in fall-related injury (10), which our trial was not powered to test.

The GP annual survey showed trends for change within the broad geographic area of the PHN. Familiarity with fall services significantly increased from 50% to over 70%, and the use of evidence-based fall prevention also increased, such as the *Stepping On Fall Prevention program*, community exercise, and Falls Clinic. While this finding is promising, more work is needed to understand and enable implementation more widely. In addition to iSOLVE activities, strong relationships with PHN staff enabled the iSOLVE resources to be included in their fall prevention Health Pathways (a guiding online resource for GP practice) (43), and they continued their commitment to GP and AHP education beyond the trial.

### 4.2 Relevance to current practice

Numerous studies have evaluated the implementation of the STEADi algorithm and workflow practices in the US (47–49), which we drew on as a basis for the iSOLVE Decision Tool. Our findings are novel in that GPs, through supported workflow practice, can change practice and engage in fall prevention, not just in screening for fall risk. The US study outcomes have focused largely on increased screening (49–52), with one study demonstrating a reduction in fall-related hospitalizations (52). In the US, some organizations have been able to embed the STEADi algorithm into electronic medical records, whereas we relied on external software, websites, or paper versions. However, a consistent finding is the importance of GPs in familiarizing themselves with the workflow (48, 50), supporting our findings of internalizing the process (15). As we found, the patient Stay Independent checklist was critical in simplifying the screening process, particularly focusing on the three key questions (fall in the past year, worried about falling, feeling unsteady) (51).

There are competing interests for GPs, and there is a shift from traditional medical approaches to a belief that a GP’s role can be to engage in fall management and prevention. Barriers related to the complexity of patient care time demands and other practical issues, such as transport, were supported in our interviews (14) and others (48, 53, 54). Yet our study, like those in the US (STEADi) (48–50), has demonstrated workflow support’s importance in mediating these challenges. Our GPs who were more confident and changed practice more often reported a more ‘proactive’ and comprehensive approach, involving discussion with their patients and less on the practical restraints.

GPs were surprised when ‘asking the question’ about a fall identified younger, less frail older adults who fall. Fall prevention actions need to address the needs of diverse groups of older people at lower, intermediate, and high risk of falls (1, 55, 56). Capturing those at intermediate risk who do not have multiple risk factors but have had a fall could have a greater longer-term preventive impact.

We articulated and reflected on what we observed as key drivers of change. These provide important lessons for wider implementation. A central part was a relevant and simple system, training, documentation, and resources. Vandervelde et al. (57), in their review of implementation strategies in fall prevention, found such technical assistance a key strategy. More work is needed for longer-term sustainability and maintenance, such as establishing partnerships, continuing commitment to fall prevention, improving communication and collaboration between multiple professionals, and ongoing attention to drivers of change (47). The barriers and facilitators of implementing fall prevention in primary care in the iSOLVE project aligned closely with those identified in the STEADI initiative (48). Since fall prevention has proven to be cost-effective (6, 58, 59) and can reduce hospitalization and mediate fall-related health services (8, 9), widespread dissemination of the iSOLVE Decision Tool and resources is warranted. The PHN is important in educating and supporting general practice and AHPs in fall prevention.

In Australia, a financial incentive for chronic disease management in general practice is available in the form of five MBS-subsidized allied health services annually. However, this scheme is underutilized for fall prevention for various reasons (3). Despite the indication of these items being effective for fall prevention in primary care (60), professionals providing services using this scheme reported inadequate reimbursement and needing to charge patients for additional expenses, insufficient numbers of items per year to provide high-quality care, and the administrative burden on the professionals to use the system (61). Sustainable reimbursement mechanisms are needed to support integrated care in the future.

Our intervention targeted changes in general practice and increased referrals to AHPs, a crucial part of fall prevention management. A novel aspect of our approach was the emphasis on upskilling AHPs, training them comprehensively, and enhancing GP awareness of the roles, range of expertise, and contact pathways for AHPs. The PHN area demonstrated high AHP engagement; however, expanding these services to meet rising demand in this and other regions will depend on fostering cross-sectorial partnerships. Further research is needed to cultivate and embed these relationships within healthcare systems (48, 57).

Referral systems for fall prevention have previously been described as fragmented (47). The reality is that fall prevention requires cross-sectorial collaboration, operating within a matrix of referral patterns and funding coalitions. Each sector plays a critical role in developing fall prevention pathways. For example, fall-related ambulance callouts could incorporate the iSOLVE patient *Stay Independent* checklist, facilitating referrals to GPs or directly them to AHPs, such as occupational therapy therapists, for home visits. Our findings showed active cross-referrals between AHPs to address patient needs (13).

In our project, the PHN acknowledged the importance of and actively participated in providing educational support for GPs and AHPs. Leadership and engagement in public health and primary care remain vital (48, 62), aligning with increasing calls for policy action to strengthen these systems (55).

### 4.3 Strengths and weaknesses

The strength of this project in exploring practice change lies in the opportunity to triangulate findings using multiple methods. Rigorous investigation of key questions was achieved through a combination of quantitative and qualitative data, supported by interviews with study participants (13–15). While surveys and interviews may be subject to social desirability bias, the primary objective was to examine the personal experiences of health professionals in their engagement with the iSOLVE program.

The inclusion of qualitative data in a pragmatic trial is a well-established approach to assessing the real-world application of an intervention (16, 17). Additionally, response rates to the annual area-wide surveys are considered reasonable (63, 64), given that the total population comprised GPs within the PHN area.

This implementation project enabled shared resources and the development of knowledge over time. It is likely that, given the pragmatic nature of the project, multiple elements of introduced bias and potential contamination occurred that influenced the embedded trial results. For example, parallel work in upskilling AHPs, trial surveys, and area-wide surveys may have raised awareness of fall prevention options. The study focused on a specific geographic area, and the findings might not be generalizable to all GP populations without further research.

## 5 Conclusion

This project has shown that GPs can successfully change practice and engage in fall prevention by equipping them with resources and strategies to become more proactive in preventing falls among their older patients. Our emphasis on facilitating evidence-based approaches, partnerships, and referral processes was crucial to this project. The project’s intent was implementation, and the goal for GPs was to implement the process routinely with all their older patients. The experimental GPs were more likely to be comprehensive in their approach, and they perceived their role in fall prevention differently. Previously, their focus might have been primarily on diagnosing and treating specific diseases. We recognized the competing demands on GPs’ time, so fall prevention processes must be relevant and easy to use in busy GP practices.

From this project, we produced a range of useful and accessible resources and a decision tool that is now freely available to general practice. By empowering GPs to proactively address fall prevention, the iSOLVE process has the potential to significantly reduce fall-related injuries. AHPs are vital in fall prevention and facilitating relationships and connectedness across primary care networks. Further, the PHN can be crucial in promoting and supporting fall prevention and facilitating referral pathways. Work is needed to implement what is learned from this and other implementation studies across primary care.

## Data Availability

The original contributions presented in the study are included in the article/Supplementary material. Further inquiries can be directed to the corresponding author/s.
