# Supplementary material for: Integrated solutions for sustainable fall prevention in primary care: a pragmatic hybrid-type 2 mixed methods implementation and effectiveness study
Source: Front Public Health. 2024 Dec 5;12:1446525. doi: 10.3389/fpubh.2024.1446525 (PMC11656318; doi:10.3389/fpubh.2024.1446525)
Supplement: Supplementary file 1 [file Data_Sheet_1.PDF]

Supplementary Material

Appendix 1 Patient Stay Independent checklist

iSOLVE Integrated SOLutions for sustainable fall preVEntion

Check your risk for falling

Check your risk for falling	Tick 'Yes' or 'No'	
**These are about your history of falls**		
I have fallen in the past year.	☐ Yes	☐ No
I am worried about falling.	☐ Yes	☐ No
**These are about balance, strength and mobility**		
I use or have been advised to use a walking stick or walker to get around safely.	☐ Yes	☐ No
Sometimes I feel unsteady when I am walking.	☐ Yes	☐ No
I steady myself by holding onto furniture when walking at home.	☐ Yes	☐ No
I need to push with my hands to stand up from a chair.	☐ Yes	☐ No
I have some trouble stepping onto a curb.	☐ Yes	☐ No
**These are about medications use**		
I am taking medication to help me sleep or improve my mood.	☐ Yes	☐ No
I am taking four or more medications.	☐ Yes	☐ No
**This is about eyesight**		
Because of my eyesight, I am finding it difficult to see where I am stepping.	☐ Yes	☐ No
**These are about other conditions associated with falls**		
I sometimes feel light-headed or dizzy.	☐ Yes	☐ No
I have foot pain that lasts for at least a day.	☐ Yes	☐ No
I often have to rush to the toilet.	☐ Yes	☐ No
I have been in hospital in the past six months.	☐ Yes	☐ No

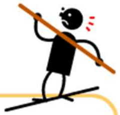

Unsteadiness and needing support are signs of poor balance or weak leg muscles, which are major reasons for falling.

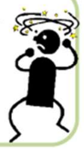

Side effects from medicines such as drowsiness and dizziness can increase your chances of falling.

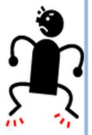

Painful feet make it difficult to walk and may cause you to stumble or trip.

Rushing to the bathroom, especially at night, increases your chances of tripping or falling.

© Clemson L, et al (2017). Integrated solutions for sustainable fall prevention in primary care, the iSOLVE project: a type 2 hybrid effectiveness-implementation design. *Implementation Science*, 12(1), 12. Reproduce freely with iSOLVE acknowledgement.

Clemson, L., Mackenzie, L., Roberts, C., Pond, D., Tan, A., Liddle, J., Sherrington, C., Lovarini, M., & Pit, S. W. (2019). Preventing Falls in Older Patients in the Community – Provider Resource. NSW Fall Prevention and Healthy Ageing Network,. Retrieved February 20 from https://fallspreventiononlineworkshops.com.au/wp-content/uploads/2018/07/Complete_Provider_Resource.pdf or https://fallspreventiononlineworkshops.com.au/resources/#gp

Appendix 2 GP fall risk assessment

iSOLVE Integrated SOLutions for sustainable fall preVEntion

GP Fall Risk Assessment

This assessment checklist can be used in conjunction with the patient's *Stay Independent* checklist.

Patient details/sticker:

Date	Ask the patient about their fall history	
	Have you had any falls in the past year?	☐ Yes ☐ No
	How many?	☐ 1 ☐ 2 or more
	Did you injure yourself?	☐ Yes ☐ No
	What do you think is the cause of the fall(s)?	
	Are you worried about falling?	☐ Yes ☐ No

Date	Risk factors	
	**Balance, Strength and Gait**	
	Using walking aid or have been advised to use walking aid	☐ Yes ☐ No
	Unsteady (e.g. feel unsteady when walking or hold onto furniture to steady when walking at home)	☐ Yes ☐ No
	Weakness, balance and mobility problems (e.g. need to push with hands to stand up from a chair, have some trouble stepping onto a curb)	☐ Yes ☐ No
	**Medications**	
	Sedatives, antidepressants or antipsychotics	☐ Yes ☐ No
	4 or more medications	☐ Yes ☐ No
	**Vision**	
	Severe impairment (macular degeneration, glaucoma, diabetic retinopathy)	☐ Yes ☐ No
	Cataract formation	☐ Yes ☐ No
	**Postural Hypotension, Light-Headedness or Dizziness**	
	A decrease in systolic BP ≥20 mm Hg or a diastolic BP of ≥10 mm Hg from lying or sitting to standing	☐ Yes ☐ No
	Light-headedness or dizziness	☐ Yes ☐ No
	**Other Medical Conditions**	
	Foot pain that lasts for at least a day	☐ Yes ☐ No
	Urge incontinence (e.g. rush to the toilet)	☐ Yes ☐ No
	Recent hospitalisation (e.g. in the past six months)	☐ Yes ☐ No
	Cognitive impairment	☐ Yes ☐ No

© Clemson L, et al (2017). Integrated solutions for sustainable fall prevention in primary care, the iSOLVE project: a type 2 hybrid effectiveness-implementation design. *Implementation Science*, 12(1), 12. Reproduce freely with iSOLVE acknowledgement.

Clemson, L., Mackenzie, L., Roberts, C., Pond, D., Tan, A., Liddle, J., Sherrington, C., Lovarini, M., & Pit, S. W. (2019). Preventing Falls in Older Patients in the Community – Provider Resource. NSW Fall Prevention and Healthy Ageing Network,. Retrieved February 20 from https://fallspreventiononlineworkshops.com.au/wp-content/uploads/2018/07/Complete_Provider_Resource.pdf or https://fallspreventiononlineworkshops.com.au/resources/#gp

Appendix 3 Tailoring interventions to fall risk factors

iSOLVE Integrated SOLutions for sustainable fall preVEntion

Tailoring Interventions to Fall Risk Factors

The following is a guide that can be used to develop a tailored management plan for your patient.

Date	Risk Assessment	Intervention/Management	Referral To/Follow-Up
	0 fall in past year + no other fall risk factor	Refer to how to fall-proof yourself in the *Stay Independent* brochure or Staying Active and On Your Feet booklet.	Community exercise with balance component.<br>Example of exercises in *Staying Active and On Your Feet* booklet (URL below).<br>NSW exercise venues: www.activeandhealthy.nsw.gov.au
	1 fall in past year, *or* worried about falling	• Group exercise with balance component (e.g. Tai Chi), or<br>• Fall prevention program (e.g. Stepping On).	Community exercise with balance component or a fall prevention program.<br>Example of exercises in *Staying Active and On Your Feet* booklet (URL below).<br>NSW exercise and Stepping On venues: www.activeandhealthy.nsw.gov.au
	Problems with balance/strength/gait	Consider individual prescription for balance and lower limb strength exercise.	Physiotherapist or exercise physiologist for exercise prescription.
	≥ 2 falls in past year, *or* Injurious falls, *or* 1 fall + unsteadiness, *or* 1 fall + recent hospitalisation	• Refer for individual prescription for balance and lower limb strength exercise.<br>• Review home safety.<br>• If required, consider referral to geriatrician or Falls Clinic.	Physiotherapist or exercise physiologist for exercise prescription.<br>Occupational therapist for home safety assessment.<br>Geriatrician or Falls Clinic, for complex care patients and those who continue to fall despite management.
	Taking sedatives, antidepressants or antipsychotics, or ≥ 4 medications	• Review indication, side effects and use of medication(s).<br>• Consider discussion with a pharmacist.	HMR pharmacist for comprehensive medication review.
	Severe vision impairment	Review home safety.	Occupational therapist for home safety assessment.
	Cataract(s)	Assess for cataract(s) surgery.	Ophthalmologist.
	Postural hypotension, dizziness, or light-headedness	Investigate underlying cause(s).	GP action: medical and/or medication management.
	Disabling foot pain	• Assess foot pain.<br>• Consider foot and ankle exercises.	FootHold Foot and Ankle exercises (www.foothold.org.au)<br>Podiatrist, physiotherapist, or exercise physiologist for exercise prescription.
	Urge incontinence	Investigate underlying cause(s).	GP action: medical and/or medication management.
	Cognitive impairment	Select falls prevention activity suited to patient's cognitive ability.	Inform referred provider(s) of patient's cognitive status.

© Clemson L, et al (2017). Integrated solutions for sustainable fall prevention in primary care, the iSOLVE project: a type 2 hybrid effectiveness-implementation design. *Implementation Science*, 12(1), 12. Reproduce freely with iSOLVE acknowledgement.

Clemson, L., Mackenzie, L., Roberts, C., Pond, D., Tan, A., Liddle, J., Sherrington, C., Lovarini, M., & Pit, S. W. (2019). Preventing Falls in Older Patients in the Community – Provider Resource. NSW Fall Prevention and Healthy Ageing Network,. Retrieved February 20 from https://fallspreventiononlineworkshops.com.au/wp-content/uploads/2018/07/Complete_Provider_Resource.pdf or https://fallspreventiononlineworkshops.com.au/resources/#gp

Appendix 4 Case study example (extracted from Preventing Falls in Older Patients in the Community – Provider Resource)

Ms Rossi

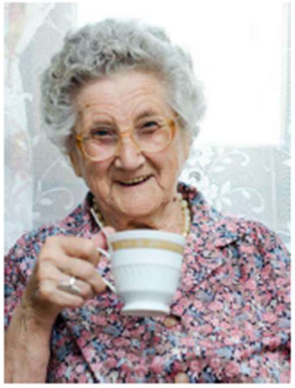

Ms Rossi is an 82 year old single woman who lives alone at home. Her 50-year-old niece visits occasionally. She was discharged from hospital about a month ago due to a urinary tract infection that has now resolved. She has come in to your practice, accompanied by her niece, for a follow-up. She is otherwise well and her diabetes condition is stable.

Ms Rossi completes the *Stay Independent* brochure in the waiting room.

Check your risk for falling	Tick 'Yes' or 'No' here	
**These are about your history of falls**		
I have fallen in the past year.	✓ Yes	☐ No
I am worried about falling.	✓ Yes	☐ No
**These are about balance, strength and mobility**		
I use or have been advised to use a walking stick or walker to get around safely.	✓ Yes	☐ No
Sometimes I feel unsteady when I am walking.	✓ Yes	☐ No
I steady myself by holding onto furniture when walking at home.	✓ Yes	☐ No
I need to push with my hands to stand up from a chair.	✓ Yes	☐ No
I have some trouble stepping onto a curb.	☐ Yes	✓ No
**These are about medications use**		
I am taking medication to help me sleep or improve my mood.	☐ Yes	✓ No
I am taking four or more medications.	✓ Yes	☐ No
**This is about eyesight**		
Because of my eyesight, I am finding it difficult to see where I am stepping.	☐ Yes	✓ No
**These are about other conditions associated with falls**		
I sometimes feel light-headed or dizzy.	☐ Yes	✓ No
I have foot pain that lasts for at least a day.	✓ Yes	☐ No
I often have to rush to the toilet.	☐ Yes	✓ No
I have been in hospital in the past six months.	✓ Yes	☐ No

Medical conditions and medications

- Type 2 Diabetes → Diaformin® (metformin)
  → Melizide® (glipizide)
- Osteoarthritis → Osteomol 665® (paracetamol)
- Hypertension → Avapro HCT® (irbesartan/hydrochlorothiazide)
- Hyperlipidemia → Lipitor® (atorvastatin)

Clemson, L., Mackenzie, L., Roberts, C., Pond, D., Tan, A., Liddle, J., Sherrington, C., Lovarini, M., & Pit, S. W. (2019). Preventing Falls in Older Patients in the Community – Provider Resource. NSW Fall Prevention and Healthy Ageing Network,. Retrieved February 20 from https://fallspreventiononlineworkshops.com.au/wp-content/uploads/2018/07/Complete_Provider_Resource.pdf or https://fallspreventiononlineworkshops.com.au/resources/#gp

Consultation

After you have conducted a standard checkup, you review Ms Rossi's answers on the *Stay Independent* brochure. Using your GP fall risk assessment checklist, you find out that she has fallen multiple times in the past year. Once she stumbled on loose pavement and fell as she was walking outside her house to collect her mail, and bruised herself. The fall reminded her of a fall a few years' back for which she ended up in hospital with a large cut on her arm. She admits that she used to be an outgoing person and enjoyed walks to the park with a few of her older neighbours. However, she is now worried about falling every time she goes out and it would be embarrassing if someone sees her fall. She is worried that she will lose her independence as she is noticing her older neighbours are starting to move into nursing homes one by one.

You explain to Ms Rossi that it is not true that the best way to prevent falls is to stay at home and limit activity. There are many things that she can do to reduce her chances of falling, such as exercise and addressing hazards at home. Using the iSOLVE tailoring interventions to fall risk factors, you think that Ms Rossi may benefit from an individual prescription for balance and lower limb strength exercise by a physiotherapist or exercise physiologist, and a home safety review by an occupational therapist.

You review her feet and find large bunions but no other lesions. She has some degree of diabetic neuropathy with poor sensation in the toes. There is no evidence of infection and the skin is intact. You previously referred Ms Rossi to a podiatrist as she is a diabetic, however, you explain that you will include in the referral letter to both the podiatrist and physiotherapist about her foot pain that may increase her chances of falling and may require further assessment.

You also check that she has adhered to your advice for annual eye checks as a diabetic. You do not think that a Home Medicines Review is necessary at this stage.

Fall prevention recommendations

- Physiotherapist/exercise physiologist for exercise prescription
- Occupational therapist for home safety review
- Referral and follow up with podiatrist/physiotherapist for foot pain

Follow-up

Ms Rossi comes back to you for a follow up appointment in a few weeks. You ask Ms Rossi how she has been doing and if she has followed any of your recommendations to prevent falls. She replies that she has been slowly working with the physiotherapist, occupational therapist and podiatrist and is feeling positive and safer. Her physiotherapist even invited her to join a group-based exercise class that is suitable for her, and she enjoys the class very much as she is also making new friends.

Appendix 5 GP survey (cluster randomised controlled trial)

**1. Over the past 3 months have you changed the way you manage your older patients who have had a fall?**	☐ Yes	☐ No
**a) If yes, please provide an example of how your practice has changed:**		

**2. What are the challenges in doing fall prevention in your practice? Please provide an example.**

**3. How confident are you that you can help your older patients reduce their risk of falling?**			
☐ Not at all confident	☐ A little confident	☐ Quite confident	☐ Very confident

4. Within your professional network, who has influence on developing your knowledge base in fall prevention in older people?

	**Not at all influential**	**A little influential**	**Quite influential**	**Very influential**
Another GP/GP registrar	☐	☐	☐	☐
Practice manager	☐	☐	☐	☐
Practice nurse	☐	☐	☐	☐
Your patient(s)	☐	☐	☐	☐
Sydney North Health Network/ former Medicare Local	☐	☐	☐	☐
Geriatrician	☐	☐	☐	☐
Physiotherapist	☐	☐	☐	☐
Exercise physiologist	☐	☐	☐	☐
Occupational therapist	☐	☐	☐	☐
Pharmacist	☐	☐	☐	☐
Optometrist/Ophthalmologist	☐	☐	☐	☐
Podiatrist	☐	☐	☐	☐
Community exercise class	☐	☐	☐	☐
Stepping On leader	☐	☐	☐	☐
Falls Clinic team	☐	☐	☐	☐
**Other (please specify):**				

5. To what extent do you believe that the following interventions are worthwhile for fall prevention in your older patients (aged 65 years and over)?

	Not worthwhile	Somewhat worthwhile	Very worthwhile
Medication review	☐	☐	☐
Cataract surgery	☐	☐	☐
Balance and lower limb strength training	☐	☐	☐
Home review for falls hazards	☐	☐	☐
Group fall prevention program	☐	☐	☐
Community exercise class	☐	☐	☐

Other interventions that are worthwhile (please specify):

**6. Are you familiar with any fall prevention services provided in your area?**	☐ Yes	☐ No	☐ Unsure

7. Have you in the past 3 months:

Managed an older patient who had a fall?	☐ Yes	☐ No	☐ Unsure
Referred an older patient to a fall prevention service provider?	☐ Yes	☐ No	☐ Unsure
Used the Active and Healthy website? http://www.activeandhealthy.nsw.gov.au	☐ Yes	☐ No	☐ Unsure

8. Have you in the past 12 months:

Sourced information on fall prevention in older people?	☐ Yes	☐ No	☐ Unsure
Sourced a list of allied health practitioners who provide fall prevention services for older people?	☐ Yes	☐ No	☐ Unsure
Undertaken educational session(s) on fall prevention in older people?	☐ Yes	☐ No	☐ Unsure

9. Thinking about your older patients (aged 65 years and over), how easy do you find organising or undertaking the following?

	I've never done this before	Very difficult	A little difficul	Somewhat easy	Very easy
Screening for fall risk factors	☐	☐	☐	☐	☐
Discussing fall prevention with your patient	☐	☐	☐	☐	☐
Managing medical risk factors for falls	☐	☐	☐	☐	☐
Conducting a medication review yourself	☐	☐	☐	☐	☐
Conducting a medication review with a pharmacist	☐	☐	☐	☐	☐
Referring to an allied health professional for fall prevention	☐	☐	☐	☐	☐
Referring to a community exercise class	☐	☐	☐	☐	☐

10. Do you use any of the following for fall prevention?

	Never	Rarely	Sometimes	Often
Health assessment for people aged 75 years and older	☐	☐	☐	☐
The Chronic Disease Management plans (formerly Enhanced Primary Care or EPC plans)	☐	☐	☐	☐

Other funding/reimbursement options that you have used:

**11. With how many of your older patients (aged 65 years and over) do you, or a member of your staff on your behalf:**				
	**None**	**Very few**	**Some**	**Most**
Assess for fall risk factors (e.g. history of falls, balance, eyesight, physical activity, etc.)	☐	☐	☐	☐
Review medications likely to contribute to fall risk	☐	☐	☐	☐
Give verbal or written advice on ways to reduce fall risks (e.g. have eyesight checked, modifying the home, be physically active, etc.)	☐	☐	☐	☐
Refer to allied health care practitioner(s) for further assessment or management of fall risks (e.g. physiotherapists, occupational therapists, pharmacists, etc.)	☐	☐	☐	☐

**12. To whom do you refer your older patients (aged 65 years and over) because they are at risk of falling?**				
	**Never**	**Rarely**	**Sometimes**	**Often**
Practice nurse	☐	☐	☐	☐
Geriatrician	☐	☐	☐	☐
Physiotherapist	☐	☐	☐	☐
Exercise physiologist	☐	☐	☐	☐
Occupational therapist	☐	☐	☐	☐
Pharmacist	☐	☐	☐	☐
Optometrist/Ophthalmologist	☐	☐	☐	☐
Podiatrist	☐	☐	☐	☐
Community exercise class	☐	☐	☐	☐
Stepping On leader	☐	☐	☐	☐
Falls Clinic team	☐	☐	☐	☐
Community nurse	☐	☐	☐	☐
Other specialist doctor	☐	☐	☐	☐
**Other (please specify profession or role):**				

**14. Do you have anything else you would like to add in regards to fall prevention in older people?**

Appendix 6 Results from AHP pre-workshop and post-workshop knowledge questions

Workshop topics	Pre-workshop		Post-workshop	
	Average percentage[a]	Range[b]	Average percentage[a]	Range[b]
**Exercise interventions**	86%	63–94%	91%	70–98%
**Lifestyle Functional Exercise**	85%	59–99%	91%	66–100%
**Foot and ankle interventions**	71%	32–94%	82%	59–100%
**Home environment intervention**	69%	17–89%	80%	48–97%
**Medication management**	73%	24–95%	73%	23–96%

[a]Average percentage: There are six knowledge questions administered pre-and post- each workshop. The percentages of attendees who achieved the correct answer for each questions were averaged across the size questions.
[b]Range: The lowest/highest percentage who achieved the correct answer for a knowledge question within each workshop.

Workshop topics	**Example low-scoring knowledge questions or questions with large percentage differences between pre- and post-**	**Pre-workshop percentage for correct answer**	**Post-workshop percentage for correct answer**
Exercise interventions	Which of the following contain exercises for improving dynamic balance?<br>a) Walking along a beam, standing reach, tandem stance<br>b) Tandem stance, stork stand, sit to stand<br>**c) Sit to stand, walking along a beam, standing reach**<br>d) Tandem stance, stork stand, standing reach	63%	70%
Lifestyle Functional Exercise	Embedding balance and strength into daily activity requires:<br>a) Doing a set number of activities for 20 minutes three times a week<br>b) Using ankle cuff weights to offer resistance<br>**c) Changing habits to incorporate a novel activity into a routine part of daily tasks**<br>d) Balance training	73%	93%
Lifestyle Functional Exercise	Which of the following contain two activities for improving dynamic balance?<br>a) Tandem walk along the hallway, heel-toe standing at the kitchen bench<br>b) Tandem stand cleaning your teeth, standing on one leg answering the phone<br>**c) Heel walking putting the dishes in the cupboard, stepping over objects then picking them up**<br>d) Tandem stand reaching into a high cupboard, one legged stand waiting in line at the supermarket	59%	66%
Foot and ankle interventions	An 82 year old woman presents with extensive bruising to her legs after a fall at the local shopping centre. Which of the following is least likely to be a fall risk factor for her:<br>a) Pain in one of her feet<br>b) Shoes with worn soles<br>**c) Ingrown nail**<br>d) Bunion	32%	59%

Foot and ankle interventions	Which of the following foot and ankle exercises has not been shown to be effective for improving foot muscle strength or flexibility?<br>a) Calf stretching<br>b) Picking up marbles with toes<br>c) Ankle circling<br>**d) Foot tapping**	43%	84%
Home environment interventions	Which of the following groups of interventions are considered to have level 1 evidence of their effectiveness in preventing falls:<br>a) Balance exercise, medication review and cataract surgery<br>b) Use of a home safety checklist, exercise groups and vision remediation<br>**c) Balance exercise, home safety for people at high risk and the Stepping On**<br>d) Home safety for people at high risk, podiatry and walking exercise	55%	71%
Home environment interventions	To be effective, home safety interventions should:<br>**a) Be conducted by an occupational therapist**<br>b) Be offered to all older people<br>c) Have a focus on education<br>d) Only be offered as part of a multi—faceted falls prevention program	17%	48%
Medication management	The strongest predictor of future falls risk is:<br>a) Polypharmacy<br>**b) Having fallen in the past year**<br>c) Diabetes<br>d) Dizziness	63%	23%
Medication management	An 82 year old woman presents with extensive bruising to her legs after a fall at the local shopping centre. Which of her medicines is most likely to have increased her chance of falling from the following list:<br>**a) Citalopram**<br>b) Warfarin<br>c) Atenolol<br>d) Metformin	24%	45%
Medication management	Which of the following is not generally associated with medications with anticholinergic activity/effects?<br>a) Dizziness<br>b) Blurred vision<br>**c) Tremor**<br>d) Confusion	78%	92%

Appendix 7 GP and patient sample per GP Practice

Practice	Group	Stratum	Number of GPs	Number of patients	Average pts per GP
**1**	Intervention	High	3	24	8.0
**2**	Control	Low	3	8	2.7
**3**	Control	Low	1	4	4.0
**4**	Intervention	High	2	24	12.0
**5**	Control	High	2	20	10.0
**6**	Intervention	High	1	20	20.0
**7**	Control	Low	2	27	13.5
**8**	Intervention	High	4[b]	27	6.8
**9**	Control	Low	1	3	3.0
**10**	Intervention	High	3	35	11.7
**11**	Control	High	3	26	8.7
**12**	Intervention	Low	1	9	9.0
**13[a]**	Control	High	3	0	0
**14**	Intervention	Low	1	4	4.0
**15**	Control	High	2	35	17.5
**16**	Control	High	2[c]	20	10.0
**17**	Intervention	High	1	35	35.0
**18**	Control	High	3	29	9.7
**19**	Control	High	2[c]	20	10.0
**20**	Intervention	High	5	35	7.0
**21**	Intervention	High	4	16	4.0
**22**	Control	High	1	15	15.0
**23**	Intervention	High	6[d]	27	4.5
**24**	Control	High	6[d]	32	5.3
**25**	Intervention	High	1	19	19.0
**26**	Control	High	6	32	5.3
**27**	Control	High	6	14	2.3
**Total**			**75[e]**	**560**	**7.5**

[a]Practice withdrew post-randomisation, prior to patient recruitment
[b]Patients recruited from 3 of the 4 participating GPs
[c]Patients recruited from 1 of the 2 participating GPs
[d]Patients recruited from 5 of the 6 participating GPs
[e]Patients recruited from 67 of the total 75 participating GPs

Appendix 8 Number of falls and Time (Days) in trial - sample of GP patients

	Experimental<br>Number patients (%)	Control<br>Number patients (%)
**Total falls over 12-month follow up**		
No falls	111 (40%)	124 (44%)
1 fall	77 (28%)	72 (25%)
2 falls	40 (15%)	32 (11%)
3–5 falls	35 (13%)	41 (14%
6–18 falls	11 (4%)	15 (5%)
Outliers	1 (<1%, n=27)	1 (<1%, n=47)
Total patients	**275**	**285**
**Time in trial for all fall outcome (Exposure)***		
< 1 week	5 (2%)	3 (1%)
< 3 months	0	3 (1%)
< 6 months	0	3 (1%)
< 9 months	1 (<1%)	1 (<1%)
< 12 months	2 (<1%)	2 (<1%)
Completed 12-months	267 (97%)	273 (96%)
Total	**275**	**285**

*See Appendix 8 - flow chart for reasons for loss to fall follow-up

Appendix 9 Area-wide GP fall prevention engagement and referrals

**Fall prevention engagement**						
	**Responses**	**2016 % (n)**	**2017 % (n)**	**2018 % (n)**	**2019 % (n)**	**Chi Sq (df) 2016 to 2019**
Familiar of fall prevention services in the local area	Yes<br>No/unsure	40 (51%)<br>38 (49%)	96 (63%)<br>57 (37%)	72 (59%)<br>50 (41%)	142 (72%)<br>56 (28%)	**χ^2 (1) = 10.41, p=.001**
Used Active and Healthy website in the past 3 months	Yes<br>No/unsure	3 (4%)<br>75 (96%)	9 (6%)<br>143 (94%)	13 (11%)<br>110 (89%)	17 (9%)<br>180 (91%)	χ^2 (1) = 1.90, p=.169
Sourced a list of allied health professional in the past 12 months	Yes<br>No/unsure	27 (34%)<br>53 (66%)	43 (28%)<br>111 (72%)	34 (28%)<br>88 (72%)	71 (36%)<br>127 (64%)	χ^2 (1) = 0.11, p=.739
Undertaken educational session(s) on fall prevention in the past 12 months	Yes<br>No/unsure	13 (16%)<br>67 (84%)	26 (17%)<br>128 (83%)	26 (21%)<br>96 (79%)	52 (26%)<br>146 (74%)	χ^2 (1) = 3.20, p=.074
**Proportion of patients engaged by the GP in fall prevention**						
	**Responses**	**2016 % (n)**	**2017 % (n)**	**2018 % (n)**	**2019 % (n)**	**Chi Sq (df) 2016 to 2019**
Assess for fall risk factors	Some/most<br>None/very few	59 (77%)<br>18 (23%)	123 (80%)<br>30 (20%)	97 (78%)<br>27 (22%)	168 (83%)<br>34 (17%)	χ^2 (1) = 1.58, p=.210
Review medications likely to contribute to fall risk	Some/most<br>None/very few	72 (92%)<br>6 (8%)	131 (86%)<br>22 (14%)	108 (87%)<br>16 (13%)	178 (88%)<br>24 (12%)	χ^2 (1) = 1.03, p=.310
Give verbal or written advice	Some/most<br>None/very few	59 (76%)<br>19 (24%)	125 (82%)<br>28 (18%)	97 (79%)<br>26 (21%)	167 (83%)<br>35 (17%)	χ^2 (1) = 1.79, p=.181
Refer to allied health professional	Some/most<br>None/very few	54 (70%)<br>23 (30%)	118 (77%)<br>35 (23%)	94 (76%)<br>30 (24%)	166 (82%)<br>36 (18%)	**χ^2 (1) = 4.85, p=.028**

Frequency of referral for fall prevention						
	**Responses**	**2016 % (n)**	**2017 % (n)**	**2018 % (n)**	**2019 % (n)**	**Chi Sq (df) 2016 to 2019**
Physiotherapist	Sometimes/ Often<br>Never/ Rarely	66 (88%)<br>9 (12%)	130 (87%)<br>19 (13%)	111 (91%)<br>11(9%)	186 (93%)<br>15 (7%)	χ^2 (1) = 1.42, p=.234
Exercise physiologist	Sometimes/ Often<br>Never/ Rarely	39 (54%)<br>33 (46%)	90 (63%)<br>52 (37%)	80 (66%)<br>41 (34%)	128 (64%)<br>72 (36%)	χ^2 (1) = 2.16, p=.142
Occupational therapist	Sometimes/ Often<br>Never/ Rarely	51 (71%)<br>21 (29%)	92 (63%)<br>53 (37%)	74 (62%)<br>45 (38%)	127 (64%)<br>72 (36%)	χ^2 (1) = 1.15, p=.283
Podiatrist	Sometimes/ Often<br>Never/ Rarely	38 (52%)<br>35 (48%)	76 (53%)<br>67 (47%)	64 (55%)<br>53 (45%)	108 (55%)<br>90 (45%)	χ^2 (1) = 0.13, p=.715
Pharmacist	Sometimes/ Often<br>Never/ Rarely	25 (35%)<br>46 (65%)	42 (29%)<br>101 (71%)	39 (33%)<br>79 (67%)	64 (33%)<br>132 (67%)	χ^2 (1) = 0.15, p=.695
Community exercise	Sometimes/ Often<br>Never/ Rarely	39 (53%)<br>34 (47%)	74 (51%)<br>71 (49%)	75(62%)<br>46 (38%)	128 (64%)<br>72 (36%)	χ^2 (1) = 2.52, p=.113
Stepping On Fall Prevention Program	Sometimes/ Often<br>Never/ Rarely	9 (13%)<br>61 (87%)	20 (14%)<br>118 (86%)	24 (21%)<br>90 (79%)	61 (32%)<br>131 (68%)	**χ^2 (1) = 9.37, p=.002**
Falls clinic	Sometimes/ Often<br>Never/ Rarely	33 (45%)<br>40 (55%)	81 (54%)<br>68 (46%)	62 (53%)<br>56 (47%)	123 (62%)<br>76 (38%)	**χ^2 (1) = 6.02, p=.014**
Geriatrician	Sometimes/ Often<br>Never/ Rarely	57 (76%)<br>18 (24%)	107 (73%)<br>40 (27%)	92 (76%)<br>29 (24%)	151 (76%)<br>48 (24%)	χ^2 (1) = 0.00, p=.983
Optometrist/ opthalmologist	Sometimes/ Often<br>Never/ Rarely	51 (70%)<br>22 (30%)	90 (63%)<br>54 (37%)	71 (60%)<br>47 (40%)	131 (66%)<br>68 (34%)	χ^2 (1) = 0.39, p=.531
Other specialist doctor	Sometimes/ Often<br>Never/ Rarely	33 (50%)<br>33 (50%)	69 (52%)<br>63 (48%)	39 (38%)<br>63 (62%)	77 (41%)<br>113 (59%)	χ^2 (1) = 1.79, p=.180
